# Dynamic changes in transposable element and gene methylation in mulberry (*Morus notabilis*) in response to *Botrytis cinerea*

**DOI:** 10.1038/s41438-021-00588-x

**Published:** 2021-07-01

**Authors:** Youchao Xin, Bi Ma, Qiwei Zeng, Wenmin He, Meiling Qin, Ningjia He

**Affiliations:** 1 State Key Laboratory of Silkworm Genome Biology, Southwest University, Beibei, Chongqing 400715 China; 2 College of Forestry, Shandong Agricultural University, Taian, Shandong 271018 China

**Keywords:** DNA methylation, Biotic

## Abstract

DNA methylation has been proposed to regulate plant stress resistance. However, the dynamic changes in DNA methylation in woody plants and their correlations with pathogenic responses are not fully understood. Here, we present single-base maps of the DNA methylomes of mulberry (*Morus notabilis*) leaves that were subjected to a mock treatment or inoculation with *Botrytis cinerea*. Compared with the former, the latter showed decreased mCG and mCHG levels and increased mCHH levels. DNA methylation inhibitors reduced resistance gene methylation levels and enhanced mulberry resistance, suggesting that the hypomethylation of resistance genes affects mulberry resistance to *B. cinerea*. Virus-induced gene silencing of *MnMET1* enhanced the expression of mulberry-resistance genes, thereby increasing the plant’s resistance to *B. cinerea*. We also found that *MITE*s play a dominant role in controlling DNA methylation levels. *MITE*s appear to be the main sources of 24-nt siRNAs that regulate gene expression through the RNA-directed DNA methylation pathway.

## Introduction

*Botrytis cinerea* is the second most common plant fungal pathogen. It is a necrotizing pathogen that destroys host cells with a series of toxic molecules and through the plant’s own defense mechanisms. This fungus infects more than 200 dicotyledonous plants, as well as some monocots^1^. It infects cash crops, such as tomatoes and petunias, and causes approximately 15–40% of fruit and flower postharvest deterioration^2^. Traditionally, this pathogen is controlled using fungicidal treatments. However, the risk of fungi developing fungicidal resistance limits the use of such treatments^3^. In addition, it is undesirable for fresh fruits and crops to contain fungicidal residues^4^. Thus, it is more feasible to increase plant stress resistance at the molecular level.

Methylation plays an important role in plant stress resistance. For instance, in *Arabidopsis*, DNA methylation controls defense responses to *Pseudomonas syringae*, and the overall destruction of methyl groups on DNA activates these responses^5^. The resistance of *Arabidopsis dcl* or *rdr* mutants to beet cyst nematodes (*Heterodera schachtii*) is stronger than that of the wild type^6^. *Tobacco mosaic virus*-induced DNA hypomethylation of LRRs is related to increased genomic rearrangements at these sites^7^. In *Medicago truncatula*, the differences in the transcript levels of the resistance gene REP1 are related to the methylation state of its promoter region, and REP1 is associated with resistance to *Erysiphe pisi*^8^.

DNA methylation is a conserved form of epigenetic marking that is related to immunity, genome stability, imprinting, and environmental responses^9,10^. In plants, DNA methylation occurs in two contexts, symmetric (CG and CHG) and asymmetric (CHH), in which H may be A, T, or C. In *Arabidopsis*, METHYLTRANSFERASE1 (MET1) maintains CG methylation (mCG), CHROMOMETHYLASE (CMT) 3 maintains CHG methylation (mCHG), and CMT2 maintains CHH methylation (mCHH)^10,11^. DOMAINS REARRANGED METHYLTRANSFERASE (DRM) 2 establishes *de novo* DNA methylation in three contexts through the RNA-directed DNA methylation (RdDM) pathway. The RdDM pathway has two main steps: siRNA biogenesis and siRNA-guided DNA methylation. The first step involves RNA polymerase (Pol) IV and DICER-LIKEs (DCLs), whereas the second step involves Pol V, ARGONAUTE4/6 (AGO4/6), and DRMs^12,13^. To balance genome methylation and maintain gene expression, plants utilize DNA demethylase to eliminate methylcytosines and replace them with unmethylated cytosine. Demeter (DME) and REPRESSOR OF SILENCING1 (ROS1) are involved in this process^9,14^.

Transposable elements (TEs) are the main sources of small RNAs in plants. Therefore, the insertion of TEs in genes is easily regulated by the RdDM pathway^15^. The activation of TEs can regulate gene expression, and the activity of TEs is regulated by DNA methylation^16^. In rice PigmS promoters, the methylation levels of TEs regulate expression in a tissue-specific manner and balance a high-resistance phenotype with low yield loss^17^. The methylation states of TEs may be key factors in regulating multiple genes simultaneously. Therefore, regulating the methylation of TEs may be an effective breeding strategy to improve desirable agronomic traits and diminish undesirable agronomic traits^18^.

There have been limited studies on the role of DNA methylation in plant–pathogen interactions, especially those in woody plants. Mulberry (*Morus* L.) is a cultivated fruit crop of the Moraceae family. Mulberry fruit is popular in Asia because of its good taste and high nutritional value. Mulberry is also rich in pharmacological components, including active compounds such as flavonoids and polysaccharides that have anti-inflammatory and hypoglycemic effects^19^. However, mulberry plants are often infected with a variety of diseases; *B. cinerea* is a main pathogen of mulberry^20^. The genome of *Morus notabilis* C.K. Schneid is relatively small (approximately 330 Mb) and has been completely sequenced^21^. More than half of the mulberry genome is composed of TEs, including *Copia* (10.44%), *Gypsy* (9.20%), *Lard* (8.59%), *Trim* (0.61%), *L1* (0.12%), *RTE* (0.29%), *PIF-Harbinger* (1.90%), *CMC* (2.37%), *hAT* (2.88%), *MuLE* (0.38%), *MITE* (13.83%), and *Helitron* (0.98%)^22,23^.

In this study, single-base resolution DNA methylation was generated from the leaves of *M. notabilis* plants mock-treated or inoculated with *B. cinerea*. DNA methylation in mulberry leaves changed dynamically after infection with *B. cinerea*. Compared with the mock samples, the inoculated samples showed decreased mCG and mCHG levels and increased mCHH levels. Many resistance-related genes showed reduced methylation, whereas metabolism-related genes showed increased methylation. The application of DNA methylation inhibitors resulted in the hypomethylation of resistance-related genes and increased resistance to *B. cinerea*, indicating that the reduced level of DNA methylation is highly significant with regard to resistance to *B. cinerea*. The expression levels of resistance genes in mulberry were correlated with the expression of *MET1*, which encodes a product that maintains mCG. The silencing of *MnMET1* induced by tobacco curly shoot virus (TbCSV) resulted in increased resistance of mulberry to *B. cinerea*. Notably, the promoter regions of many resistance genes were hypomethylated, which may explain the epigenetic regulation of resistance to *B. cinerea* in mulberry. In addition, although the *MITEs* in mulberry account for only 13.2% of the genome, they are the most important TE-regulating genes expressed through the RdDM pathway, which is the source of 24-nt siRNAs. In summary, our study revealed for the first time the DNA methylation dynamics of a woody plant genome in response to *B. cinerea*.

## Results

### DNA methylome of mulberry leaves

**Fig. 1:**
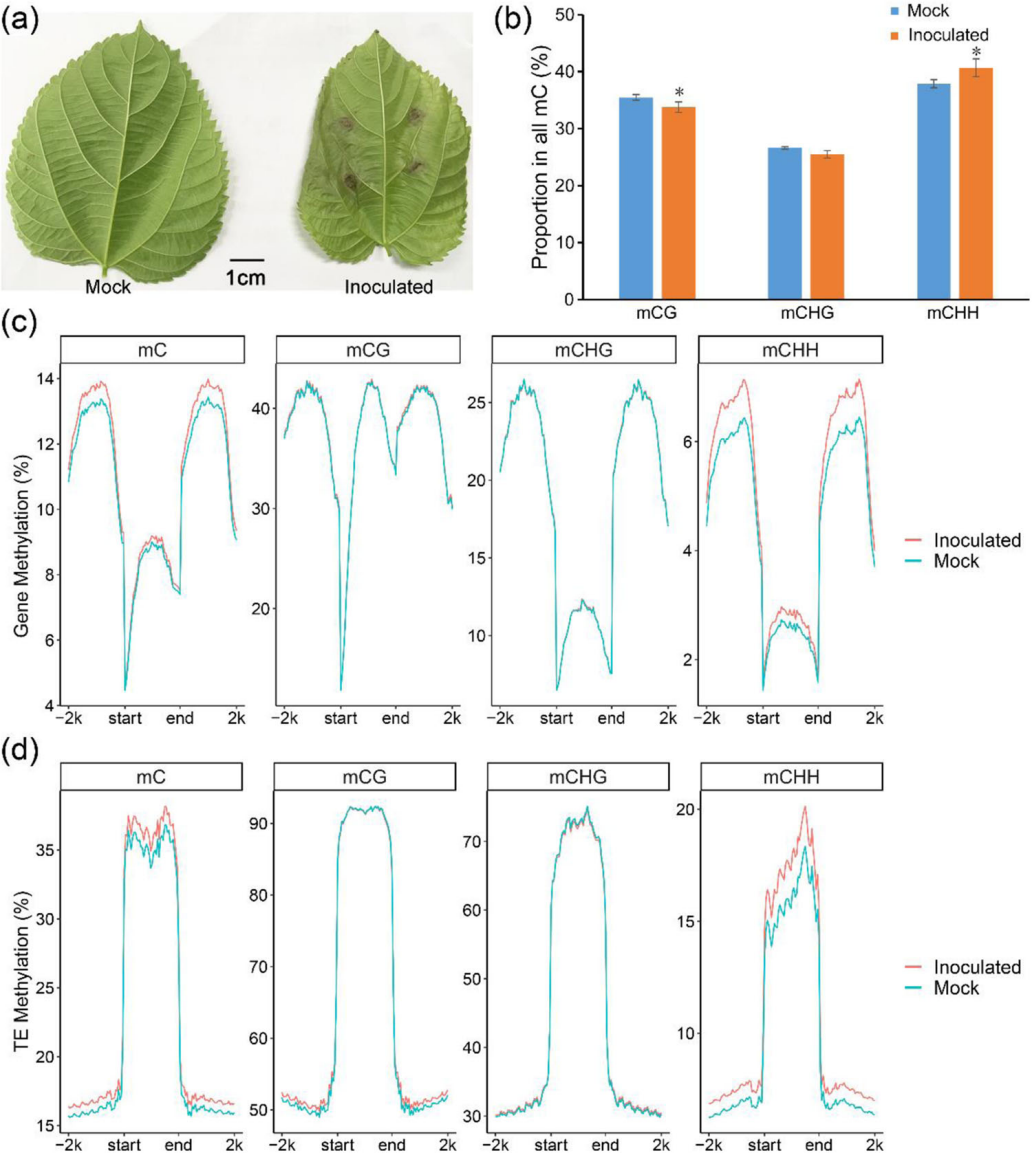
Characterization of mulberry methylomes. **a** Photographs of mock-treated (Mock) and *B. cinerea*-inoculated (Inoculated) mulberry leaves. **b** Proportions of mCG, mCHG, and mCHH in all the methylcytosine in Mock and Inoculated leaves; error bars indicate SDs, *n* = 3 (*P-value < 0.05, two-tailed *t*-test). Levels of mC, mCG, mCHG, and mCHH surrounding the upstream, gene body, and downstream regions of the genes (**c**) and TEs (**d**) in two samples. The mean values of three biological replicates are shown

To analyze mulberry methylomes, we conducted whole-genome bisulfite sequencing and generated single-base DNA methylation maps for mulberry leaves. For the mock treatment (mock) and the *B. cinerea* inoculation treatment (inoculated), DNA from three biological leaf replicates was sequenced (Fig. 1a). The genome of *M. notabilis* is approximately 330 Mb (2*n* = 14). Each sequencing library produced approximately 100 M paired-end reads (100–500 bp), covering approximately 80% of the genome. For each library, the conversion rate was approximately 99.5% (Table S1). The sequencing depth reached 50×, indicating that our sequencing data were of sufficient quality for further analyses.

Averaged across the genome, the percentages of mCG, mCHG, and mCHH in the total mC sites were 35.5, 26.6, and 37.9%, respectively, in the mock samples and 33.8, 25.5, and 40.7%, respectively, in the inoculated samples (Fig. 1b). Compared with the mock samples, the inoculated samples showed significantly decreased mCG, slightly decreased mCHG, and significantly increased mCHH levels. Thus, dynamic methylation changes occurred in mulberry in response to *B. cinerea* infection.

Next, we analyzed the average DNA methylation levels of genes and TEs (Fig. 1c, d). In genes, the mCG level was higher in both the gene bodies and their flanking regions, similar to previous findings in soybean^24^ and sugar beet^25^, while the mCHG and mCHH levels were lower in the gene bodies but higher in the flanking regions. Adjacent to the genes, the levels of mCG, mCHG, and mCHH were low. However, as the distance from the genes increased, so did the levels of mCG, mCHG, and mCHH. In the TEs, the levels of mCG, mCHG, and mCHH were much higher in the gene bodies than in the flanking regions, similar to previous findings in orange^26^ and strawberry^27^. Because TEs play important roles in genome evolution and gene regulation, we analyzed the individual methylation patterns of different TE superfamilies, including retroTEs (*Copia*, *Gypsy*, *Lard*, *Trim*, *L1*, and *RTE*) (Fig. S1) and DNA TEs (*PIF-Harbinger*, *CMC*, *hAT*, *MuLE*, *MITE*, and *Helitron*) (Fig. S2). The methylation model of *MITE* was consistent with that of a TE, indicating that *MITE* plays a leading role in TE methylation (Fig. 1d and Fig. S2). Interestingly, compared with other TEs, *MITE* showed significantly higher mCHH levels, which is indicative of the potential role of asymmetric methylation in *MITE* silencing.

### Expression profiles of genes related to DNA methylation and the RdDM pathway

**Fig. 2:**
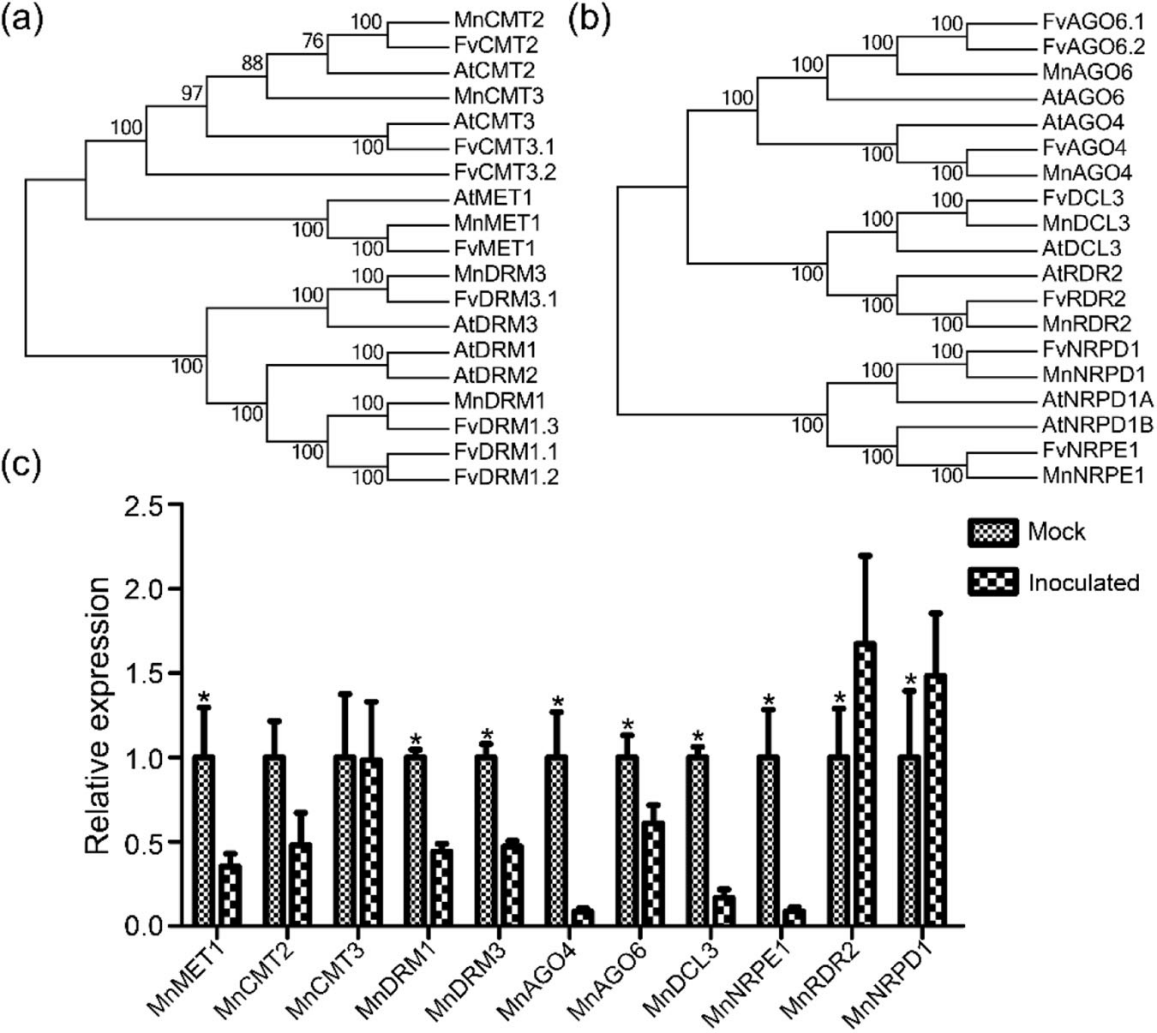
Expression of genes involved in DNA methylation. Phylogenetic analyses of DNA methyltransferase (**a**) and RdDM (**b**) genes in mulberry, strawberry, and *Arabidopsis*. The accession numbers of the CDSs are as follows: AtDRM1 (AT5G15380), AtDRM2 (AT5G14620), AtDRM3 (AT3G17310), AtMET1 (AT5G49160), AtCMT2 (AT4G19020), AtCMT3 (AT1G69770), FvDRM1.1 (gene05866), FvDRM1.2 (gene06047), FvDRM1.3 (gene28439), FvDRM3.1 (gene17910), FvMET1 (gene13037), FvCMT2 (gene13664), FvCMT3.1 (gene10077), FvCMT3.2 (gene15171), MnCMT2 (KE346101.1), MnCMT3 (KE343837.1), MnMET1 (KE344409.1), MnDRM3 (KE344683.1), MnDRM1 (KE345913.1), AtAGO4 (AT2G27040), AtAGO6 (AT2G32940), AtDCL3 (AT3G43920), AtNRPD1A (AT1G63020), AtNRPD1B (AT2G40030), AtRDR2 (AT4G11130), FvAGO4 (gene07657), FvAGO6.1 (gene16926), FvAGO6.2 (gene16928), FvDCL3 (gene15481), FvNRPD1 (gene32373), FvNRPE1 (gene14287), FvRDR2 (gene32159), MnAGO4 (KE344194.1), MnAGO4 (KE346072.1), MnDCL3 (KE344662.1), MnNRPD1 (KE345823.1), MnNRPE1 (KE344454.1), and MnRDR2 (KE345786.1). **c** qRT-PCR analyses of DNA methylation genes. All the expression levels were normalized to the expression of the mulberry actin gene. Error bars indicate SDs, *n* = 3 (*P-value < 0.05, two-tailed *t*-test)

Genomic DNA methylation can be maintained through the interaction of DNA methylation with DNA demethylation processes. To analyze the mechanisms of dynamic DNA methylation in mulberry resistance to *B. cinerea*, we first detected the expression levels of DNA demethylase genes, including *MnDME* and *MnDML*. Their transcript levels were not significantly different between the mock and inoculated samples (Fig. S3). In *Arabidopsis*, mCG, mCHG, and mCHH are maintained by MET1, CMT3, and CMT2, respectively, and all three can be *de novo* methylated by DRMs through the RdDM pathway^9,10^. To further understand why DNA methylation patterns change, we compared the transcript levels of genes involved in DNA methylation and the RdDM pathway between the mock and inoculated samples. Five DNA methyltransferase genes were identified in the mulberry genome: *MnCMT2*, *MnCMT3*, *MnMET1*, *MnDRM1*, and *MnDRM3* (Fig. 2a). Compared with those in the mock samples, the transcript levels of all these genes, except *MnCMT3*, in the inoculated samples were lower (Fig. 2c). The downregulation of *MnDRM1* and *MnDRM3* suggested that RdDM activity might decrease during *B. cinerea* infection. To test this hypothesis, we detected the transcript levels of RdDM-related genes. RdDM consists of two stages: siRNA production, which requires Pol IV, RDR2, and DCL3, and siRNA-mediated DNA methylation, which requires Pol V, AGO4/6, and DRMs. We found genes encoding the largest subunits of Pol IV (*MnNRPD1*) and Pol V (*MnNRPE1*), as well as *MnRDR2*, *MnDCL3*, *MnAGO4*, and *MnAGO6*, in the mulberry genome (Fig. 2b). Compared with those in the mock samples, the transcript levels of *MnRDR2* and *MnNRPD1* in the inoculated samples significantly increased, whereas the transcript levels of the other genes significantly decreased (Fig. 2c).

### Analyses of siRNA expression and DNA methylation

**Fig. 3:**
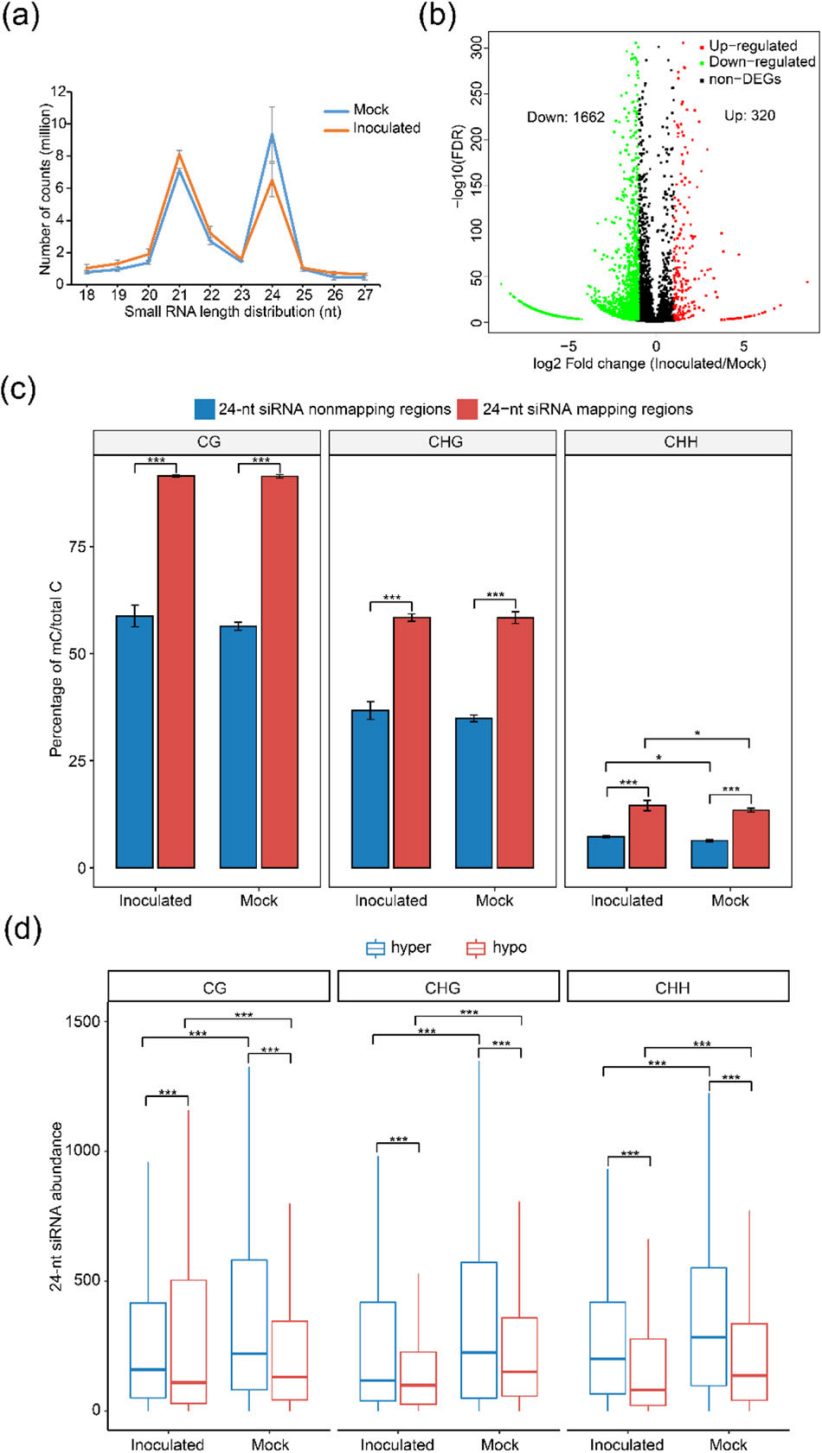
DNA methylation and 24-nucleotide siRNAs. **a** Size distribution of the sequenced small RNAs in mulberry. Three biological replicates are shown. **b** Volcano plot indicating the up- and downregulated 24-nt siRNAs between the two groups. **c** DNA methylation levels in mapped regions with 24-nt siRNAs compared with those in mapped regions without 24-nt siRNAs in mock-treated (Mock) and *B. cinerea*-inoculated (Inoculated) mulberry leaves. Error bars indicate SDs, *n* = 3 (*P-value < 0.05, ***P-value < 0.001, two-way ANOVA). **d** Abundance of 24-nt siRNAs located in the CG, CHG, and CHH hypermethylated and hypomethylated regions (***P-value < 0.001, two-way ANOVA)

The RdDM pathway is usually dependent on 24-nt siRNAs. Therefore, we studied the expression profiles of small RNAs in the mock and inoculated samples using high-throughput sequencing. Among the small RNAs, lengths of 21-nt and 24-nt were the most abundant in the mulberry samples (Fig. 3a). In the mock samples, the latter was the most abundant type of small RNA, whereas, in the inoculated samples, the former was the most abundant. Compared with the mock samples, the inoculated samples had significantly lower amounts of 24-nt small RNAs. Next, the expression levels of 24-nt siRNAs were compared between the mock and inoculated samples (Fig. 3b). In total, 1,982 differentially expressed 24-nt siRNAs (adjusted *P*-value < 0.01), including 320 upregulated 24-nt siRNAs and 1,662 downregulated 24-nt siRNAs, were identified between the inoculated and mock samples.

**Fig. 4:**
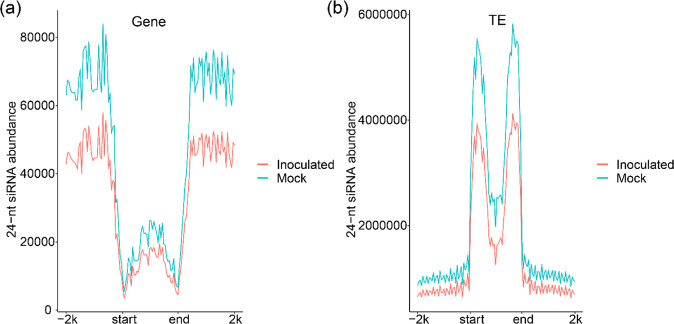
Distribution of 24-nt siRNAs. The 24-nt siRNA distributions on the gene bodies and flanking regions (**a**) and TEs (**b**) of mock-treated (Mock) and *B. cinerea*-inoculated (Inoculated) mulberry leaves. The mean values of three biological replicates are shown

To further explore the correlations between 24-nt siRNAs and methylation, we compared the average methylation levels between regions with and without mapped 24-nt siRNAs in the mock and inoculated samples (Fig. 3c). The levels of mCG, mCHG, and mCHH were significantly greater in the regions with mapped siRNAs than in the regions without mapped siRNAs. Compared with those in the mock samples, the mCHH levels in the inoculated samples were significantly greater. Interestingly, in the mock samples, more 24-nt siRNAs were present in CG-, CHG-, and CHH-hypermethylated regions than in hypomethylated regions (Fig. 3d), whereas fewer 24-nt siRNAs were present in CG-hypermethylated regions than in hypomethylated regions in the inoculated samples. Compared with those in the mock samples, the abundance levels of 24-nt siRNAs were significantly reduced in all the inoculated samples, except for the CG hypomethylation regions. We analyzed the distributions of 24-nt siRNAs on gene bodies, TE bodies, and their flanking regions (Fig. 4a, b). The 24-nt siRNAs of mulberry were preferentially located on the flanking regions of genes but were more abundant on TE bodies than on their flanking regions. Interestingly, 24-nt siRNAs were concentrated mainly at the ends of TE bodies. Compared with the mock samples, the inoculated samples had fewer 24-nt siRNAs on gene promoters, 3′ regulatory regions, and TE bodies. We further analyzed the distribution of 24-nt siRNAs in each TE superfamily (Figs. S4 and S5). The 24-nt siRNA distribution on TEs was concentrated mainly in the *MITE* superfamily, indicating that *MITE* plays an important role in generating 24-nt siRNAs.

### Differentially methylated regions and responses to *B. cinerea* stress

**Fig. 5:**
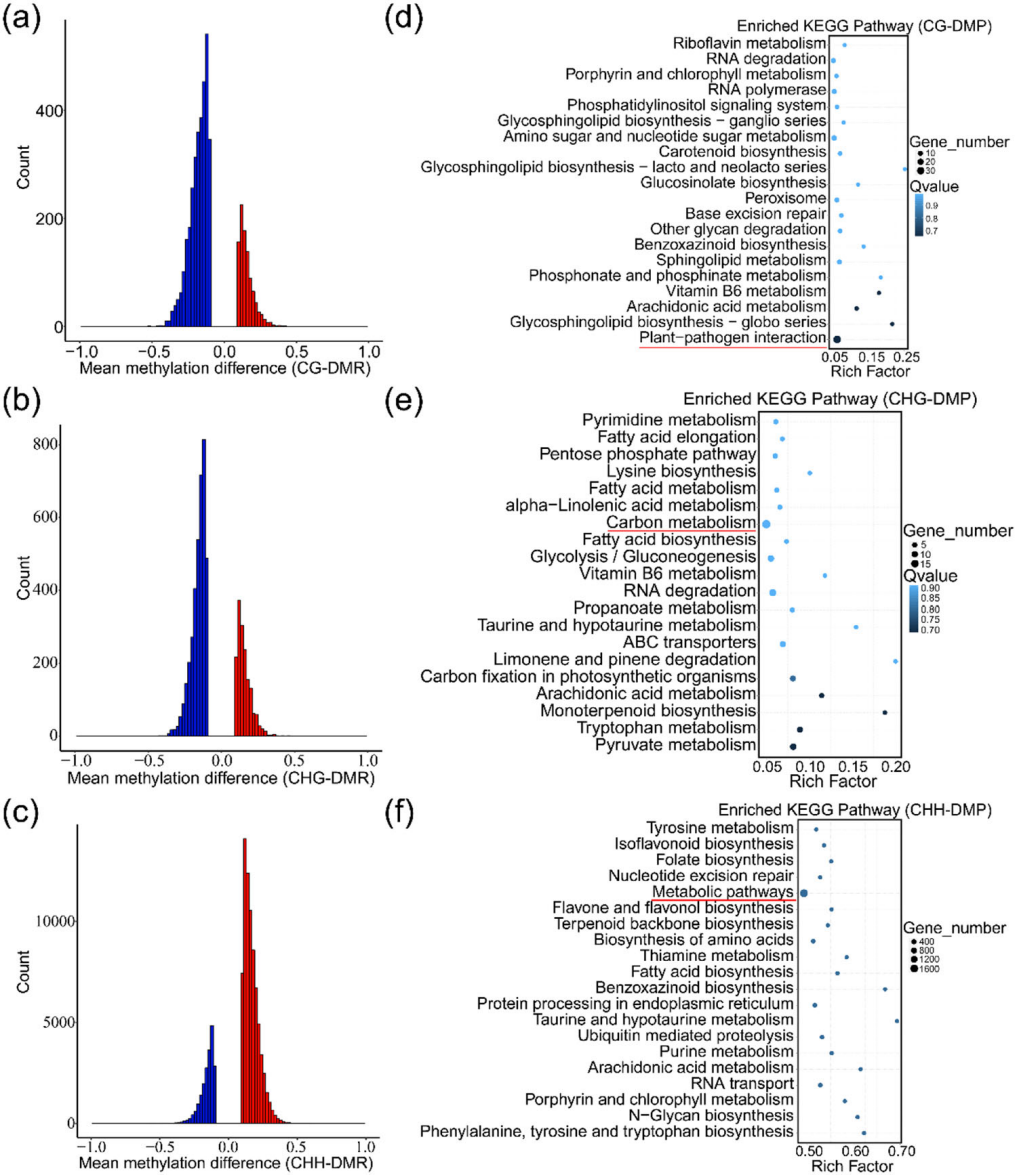
DMR and KEGG pathway enrichment analyses of DMPs between mock-treated (Mock) and *B. cinerea*-inoculated (Inoculated) mulberry leaves. Hypo- and hyperdifferentially methylated region counts of CG (**a**), CHG (**b**), and CHH (**c**). Enriched KEGG pathways of the differentially methylated promoters in CG (**d**), CHG (**e**), and CHH (**f**). The most enriched pathways are underlined in red

To explore the potential functions of methylation in the responses to *B. cinerea*, we analyzed differentially methylated regions (DMRs) in the genome between mock and inoculated mulberry plants (Fig. 5). Compared with mock samples, inoculated samples had 104,957 DMRs, of which 4,288 were mCG, 5,412 were mCHG, and 95,257 were mCHH (Fig. 5a–c). Among all the DMRs, 77.12% were hypo-DMRs of mCG, 70.12% were hypo-DMRs of mCHG, and 21.25% were hypo-DMRs of mCHH. Most of the CG and CHG DMRs were hypo-DMRs, and most of the CHH DMRs were hyper-DMRs, consistent with previous results (Fig. 1b). In addition, we identified some of the DMRs (Fig. S6).

Methylation in promoter regions regulates gene expression, and promoter methylation is negatively correlated with gene expression. To analyze the changes in promoter methylation in mulberry under *B. cinerea* stress, we searched for differentially methylated promoters (DMPs) situated in the DMRs. The search revealed 976 (811 hypo-DMPs), 1,003 (774 hypo-DMPs), and 12,779 (2,842 hypo-DMPs) DMPs of mCG, mCHG, and mCHH, respectively. To understand the functions of DMPs, Kyoto Encyclopedia of Genes and Genomes (KEGG) pathway enrichment analysis was performed to identify the pathways enriched for genes with DMPs (Fig. 5d–f). The KEGG pathways showed that plant–pathogen interaction (87.50% hypo-DMPs) was the main category of genes with CHG−DMPs, carbon metabolism (73.68% hypo-DMPs) was the main category of genes with CHG-DMPs, and metabolic pathways (21.83% hypo-DMPs) were the main category of genes with CHH−DMPs (Fig. S7a). These results indicated that mCG and mCHG may participate in responses to *B. cinerea* by enhancing the expression of genes related to disease resistance and carbon metabolism and that mCHH may be involved in these responses by inhibiting the expression of genes related to metabolic pathways.

Compared with the mock samples, the inoculated samples had 6,273 differentially methylated genes (DMGs) consisting of 954 (599 hypo-DMGs), 625 (430 hypo-DMGs), and 4,694 (1,117 hypo-DMGs) DMGs of mCG, mCHG, and mCHH, respectively, located in DMRs (Fig. S8). Interestingly, the KEGG pathway analysis showed that metabolic pathways contained the most genes with differential CG, CHG, and CHH methylation levels between the mock and inoculated samples. In the metabolic pathway category, hypo-DMGs accounted for 52.90, 71.72, and 26.84% of the total DMGs of mCG, mCHG, and mCHH, respectively (Fig. S7b). This suggests that the dynamic differential methylation of genes related to metabolic pathways may play a key role in the response to *B. cinerea*. We performed a KEGG enrichment analysis of the differentially expressed genes (DEGs) between inoculated and mock samples identified from the transcriptome data (Fig. S9) and found that the DEGs were associated mainly with plant–pathogen interaction, MAPK signaling, and plant hormone signal transduction. These findings suggest that all of these pathways are involved in responses to *B. cinerea* stress in mulberry.

### Gene and TE DMRs

**Fig. 6:**
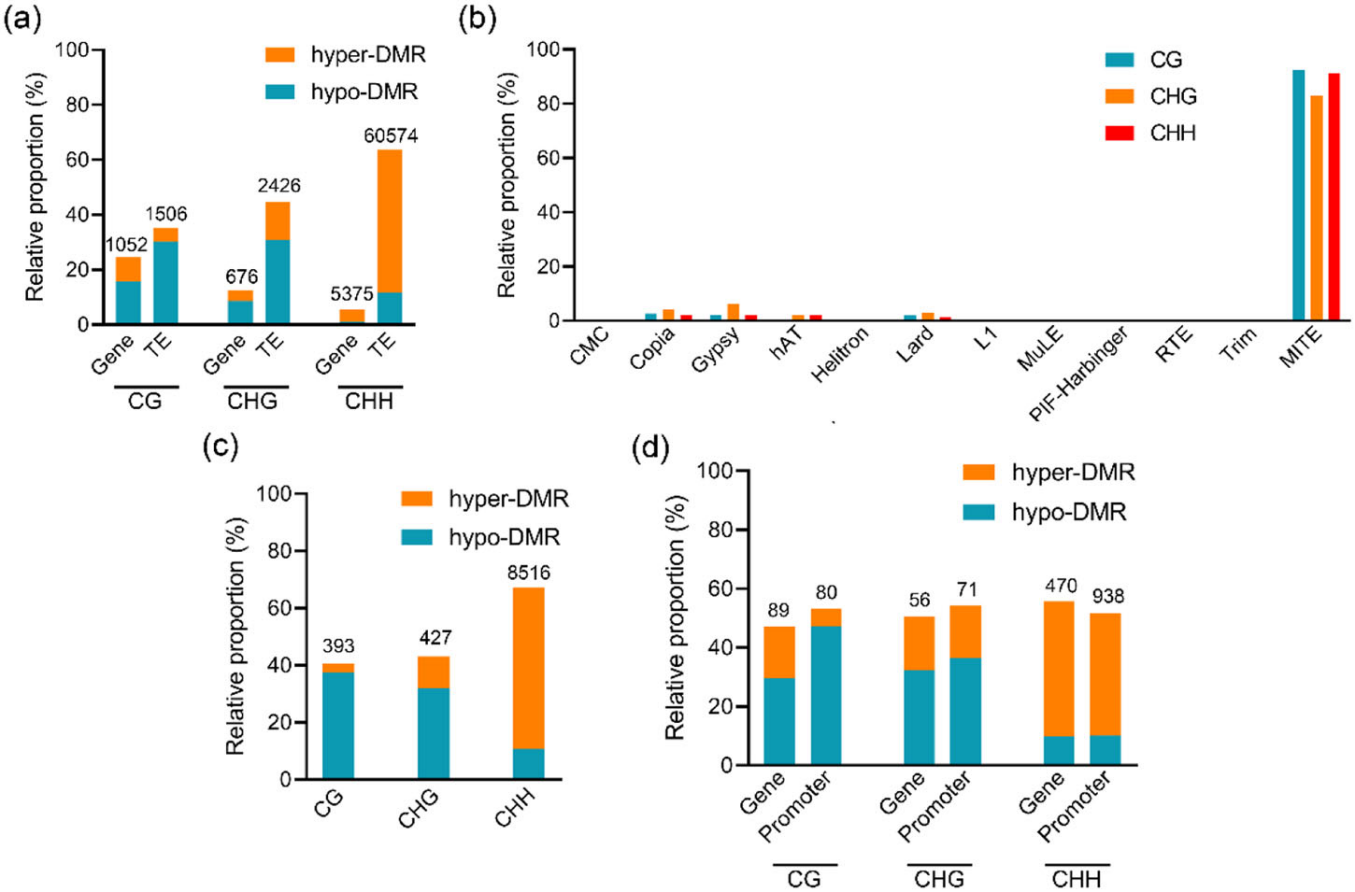
Differentially methylated regions of genes and TEs. **a** The relative proportions of hypo- and hyperdifferentially methylated regions that overlapped with genes and TEs. **b** The relative proportions of differentially methylated regions in CG, CHG, and CHH that overlapped with TEs in each superfamily. **c** The relative proportions of differentially methylated promoters in CG, CHG, and CHH that overlapped with TEs. **d** The proportions of transcripts that increased with decreased methylation and decreased with increased methylation. The number at the top of the figure represents the absolute number of DMRs

We analyzed the proportions of CG, CHG, and CHH DMRs that overlapped with genes and TEs (Fig. 6a). The proportions of DMRs, especially CHG and CHH, had significantly greater overlaps with TEs than with genes, and 63.59% of the CHH hyper-DMRs overlapped TEs. Further analyses showed that the DMRs overlapped mainly with the *MITE* superfamily of TEs (Fig. 6b). Gene promoter regions affect transcriptional regulation, and differences in promoter methylation affect transcriptional expression. The proportions of CG, CHG, and CHH DMPs that overlapped TEs (Fig. 6c) were 40.60, 43.13, and 67.22%, respectively. Next, we analyzed the distributions of DMPs that overlapped with each TE superfamily (Fig. S10). DMPs overlapped mainly with the *MITE* superfamily. Furthermore, we calculated the proportions of methylation changes in gene and promoter regions that were negatively correlated with changes in gene transcription levels (the proportions of transcripts that increased with decreased methylation and decreased with increased methylation) (Fig. 6d). Approximately 50% of methylation changes were negatively correlated with changes in gene transcription levels. This indicated that the methylation of both the gene body and the promoter region affects gene expression.

### Effects of DNA methylation on gene expression

**Fig. 7:**
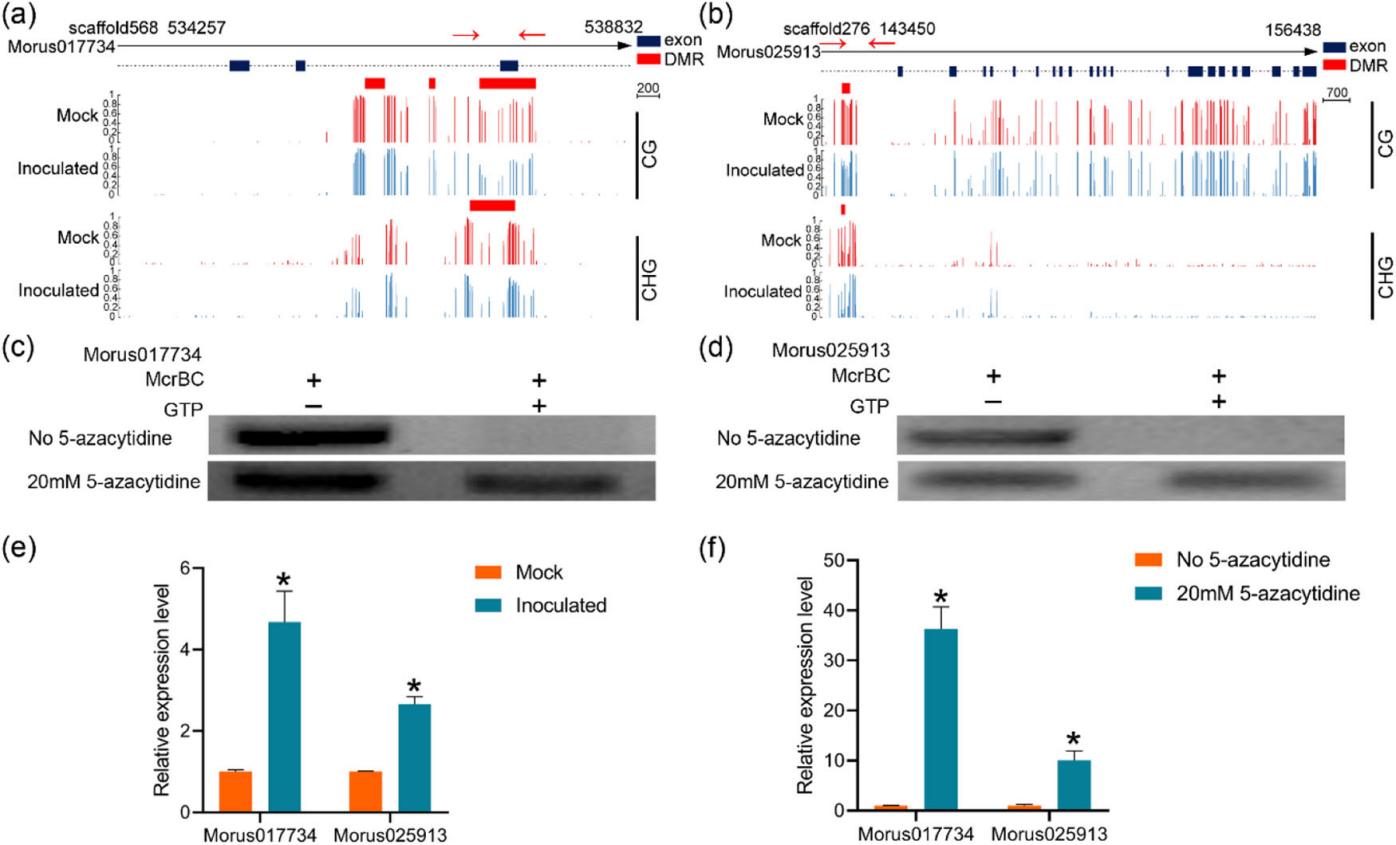
Effects of DNA methylation on the expression of mulberry-resistance genes. DNA methylation surrounding *Morus017734* (**a**) and *Morus025913* (**b**) in mock-treated (Mock) and *B. cinerea*-inoculated (Inoculated) mulberry leaves. The red rectangles represent DMRs. The red arrows represent the regions verified by McrBC-PCR. McrBC-PCR analyses of *Morus017734* (**c**) and *Morus025913* (**d**). + and − indicate the presence and absence of GTP, respectively. **e** The expression of *Morus017734* and *Morus025913* in mock-treated (Mock) and *B. cinerea*-inoculated (Inoculated) mulberry leaves. **f** The expression of *Morus017734* and *Morus025913* in mulberry leaves treated with and without 5-azacytidine. All the expression levels were normalized to the expression of the mulberry actin gene. Error bars indicate SDs, *n* = 3 (*P-value < 0.05, two-tailed *t*-test)

To determine how DNA methylation affects mulberry resistance, we applied the DNA methylation inhibitor 5-azacytidine to mulberry leaves. To confirm that the inhibitor treatment changed the DNA methylation of resistance genes in mulberry, we used methylation-sensitive McrBC-PCR to detect the DNA methylation levels of two resistance-related genes, *Morus017734* (DMG), encoding the CYSTM domain-containing protein, and *Morus025913* (DMP), encoding an LRR receptor-like serine/threonine-protein kinase (Fig. 7a, b). The enzyme McrBC, which cuts methylated DNA but not unmethylated DNA, was used to cut the genomic DNA. Then, McrBC-PCR was conducted using the digested DNA as the template. The methylation-sensitive McrBC-PCR analyses showed that both genomic regions were highly methylated, consistent with the whole-genome bisulfite sequencing results. The DNA methylation levels were decreased significantly in the 5-azacytidine-treated samples (Fig. 7c, d). To verify the effects of decreased methylation levels on gene expression, we determined the expression levels of these two genes. Compared with the mock samples, the inoculated samples showed significantly decreased methylation levels in the two genomic regions and the significant upregulation of these two genes (Fig. 7e). In the 5-azacytidine-treated samples, the two genes were also significantly upregulated (Fig. 7f). Next, to explore whether inhibiting methylation enhanced mulberry resistance to *B. cinerea*, mulberry leaves treated with or without 5-azacytidine were inoculated with *B. cinerea* (Fig. S11). The untreated leaves developed severe necrotic lesions by 3 days postinoculation, while those treated with 5-azacytidine developed mild necrotic lesions (Fig. S11a). A quantitative analysis showed that the growth of *B. cinerea* was inhibited in the 5-azacytidine-treated leaves but not in the untreated leaves (Fig. S11b).

### *MnMET1* silencing in mulberry enhanced resistance to *B. cinerea*

**Fig. 8:**
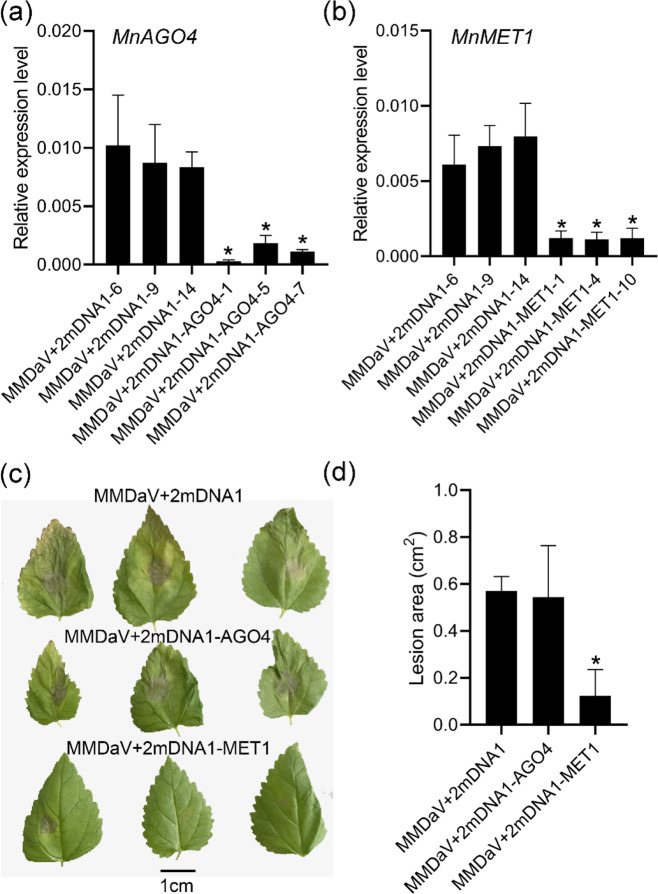
Silencing of *MnMET1* in mulberry leaves resulted in increased resistance. Relative expression levels of *MnAGO4* (**a**) and *MnMET1* (**b**) in mulberry leaves collected from three replicates injected with MMDaV+2mDNA1, MMDaV+2mDNA1-AGO4, and MMDaV+2MDNA1-MET1. All the expression levels were normalized to the expression of the mulberry actin gene. Error bars indicate SDs, *n* = 3 (*P-value < 0.05, two-tailed *t*-test). **c** Mulberry leaves inoculated with *B. cinerea* were photographed at 3 days after inoculation. **d** Quantitative analyses of the resistance to *B. cinerea* of mulberry leaves injected with MMDaV+2mDNA1, MMDaV+2mDNA1-AGO4, and MMDaV+2MDNA1-MET1

Because the 24-nt siRNA abundance and mCG levels in the promoter regions of resistance-related genes were reduced in the inoculated samples, we conducted virus-induced gene silencing (VIGS) of *MnAGO4* and *MnMET1* independently in mulberry. Phytoene desaturase (PDS) was used as a positive control (Fig. S12). The colors of plants injected with MMDaV+2mDNA1-*MnPDS* (at 14 days after VIGS treatment) were significantly paler than those of plants injected with the control construct, MMDaV+2mDNA1 (Fig. S12a). Compared with the control plants (injected with MMDaV+2mDNA1), the plants treated with MMDaV+2mDNA1-*MnPDS* showed silencing of PDS expression in their second leaves, as confirmed by gene-specific qRT-PCR analysis. The significantly decreased transcript level of *MnPDS* confirmed that its expression was effectively inhibited by VIGS (Fig. S12b). Next, the transcript levels of *MnAGO4* and *MnMET1* were detected in the MMDaV+2mDNA1 control group, as well as in the MMDaV+2mDNA1-*MnAGO4* and MMDaV+2MDNA1-*MnMET1* treatment groups. We detected significant reductions in the transcript levels of *MnAGO4* in the MMDaV+2mDNA1-*MnAGO4* treatment group and of *MnMET1* in the MMDaV+2MDNA1-*MnMET1* treatment group (Fig. 8a, b). To evaluate the resistance of mulberry to *B. cinerea* after the silencing of *MnAGO4* and *MnMET1*, the leaves of gene-silenced and control mulberry lines were inoculated with *B. cinerea*. The control leaves developed severe dry necrosis by 3 days post inoculation, whereas the leaves of *MnAGO4*-silenced plants showed slightly milder symptoms and those of *MnMET1*-silenced plants developed only mild necrotic lesions (Fig. 8c). A quantitative analysis showed that compared with the control plants, those with silenced *MnAGO4* expression did not show significantly inhibited *B. cinerea* growth, but those with silenced *MnMET1* expression showed significantly inhibited *B. cinerea* growth (Fig. 8d). In addition, *Morus002632*, which encodes the resistance-related LRR receptor-like serine/threonine-protein kinase, was expressed in *MnMET1*-silenced plants but not in the control or *MnAGO4*-silenced plants (Fig. S12c). Further analyses of methylation in the promoter region of *Morus002632* showed that the methylation level in the *MnMET1*-silenced plants was decreased compared with that in the control and *MnAGO4*-silenced plants (Fig. S12d). This indicated that the silencing of *MnMET1* reduced methylation and enhanced the expression of resistance genes in mulberry, resulting in enhanced resistance to *B. cinerea*.

To further verify the role of *MnMET1* in mulberry resistance, *MnMET1*was transiently overexpressed (Fig. S13). We detected a significant increase in the transcript level of *MnMET1* in the pLGNL-*MnMET1* treatment group (Fig. S13a). To evaluate the resistance of mulberry to *B. cinerea* after transient *MnMET1* overexpression, transiently overexpressed and control mulberry leaves were inoculated with *B. cinerea* (Fig. S13b). The control leaves showed mild necrotic lesions 2 days after inoculation, whereas the leaves of plants transiently overexpressing *MnMET1* showed more necrotic lesions. A quantitative analysis showed that, compared with that of control plants, the resistance of plants transiently overexpressing *MnMET1* to *B. cinerea* was significantly reduced (Fig. S13c).

## Discussion

Genome-wide methylome analyses performed through bisulfite sequencing have been applied to diverse plants, such as *Arabidopsis*^28^, rice^29^, tomato^30^, apple^31^, poplar^32^, and spruce^33^. Here, cytosine methylation in mulberry was analyzed through genome-wide bisulfite sequencing. We compared the dynamic methylation (hypomethylated and hypermethylated sites) between mock-inoculated and *B. cinerea*-inoculated plants. The proportions of mCG, mCHG, and mCHH in the total mC sites were 35.5, 26.6, and 37.9%, respectively, in the mock samples. In Poaceae, mCHH is usually depleted in deep heterochromatin and abundant in gene-rich regions^34^. In contrast, in mulberry, mCHH was less abundant in gene-rich regions and more abundant in TE-rich regions (Fig. 1c, d). TEs account for more than half of the mulberry genome^22,23^. Among all the TEs, *MITE* plays a dominant role in CHH methylation; this may be related to its high copy number or high activity. In the mulberry genome, *MITE* is the most abundant TE.

In the endosperm of *Arabidopsis* and rice, CG demethylation is accompanied by local CHH hypermethylation^35,36^. In tomato, global DNA hypomethylation during development is accompanied by CHH hypermethylation of TEs^30^. In this study on mulberry, compared with mock samples, inoculated samples showed dynamic changes in methylation, with significantly decreased mCG levels, slightly decreased mCHG levels, and significantly increased mCHH levels (Fig. 1b). The promoter regions of genes related to resistance were generally hypomethylated, whereas those of genes associated with metabolism were generally hypermethylated (Fig. 5). Hypomethylation and hypermethylation are beneficial for controlling gene expression in mulberry under *B. cinerea* stress. Hypermethylation reduces gene transcription, which decreases energy consumption. This means that more energy can be directed to the pathogen defense response. In contrast, the hypomethylation of resistance-related genes increases their expression, resulting in rapid resistance responses to *B. cinerea*. The activation of TEs is determined mainly by their DNA methylation levels^16^. Genome-wide hypomethylation owing to biological or nonbiological stress activates TEs and increases their fluidity in disease-related genes, thereby regulating gene expression^5,37–39^. In mulberry, the differential methylation of CGs was detected in similar proportions in gene and TE regions, while the differential methylation of CHGs and CHHs was detected mainly in TE regions (Fig. 6a). In particular, the differential methylation of CHHs was detected mainly in TE regions, indicating that CHH methylation plays a key role in inhibiting TE activity (Fig. 6b). This also suggests that mulberry TEs may play important roles in regulating gene expression through the RdDM pathway. Transposable elements in promoters regulate gene expression by changing their DNA methylation states. Analyses of the methylation states of TEs in mulberry showed that more than 80% of the differences in methylation occurred in *MITE*s (Fig. S10). These *MITE*s tended to be inserted near genes^23^, indicating that *MITE* may be the most important type of TE regulating gene expression through the RdDM pathway.

DNA methylation is regulated by DNA methylation and demethylation. In *Arabidopsis*, DNA demethylation is catalyzed by the REPRESSOR OF SILENCING1 family^9^. These proteins remove mC bases and cleave the DNA backbone, which is then filled by an unmethylated cytosine. In *Arabidopsis*, DME contributes to DNA demethylase activity and disease responses^40^, and TE sequences in the promoter regions of resistance-related genes are targets of DNA demethylases^41,42^. Changes in gene promoter methylation in mulberry occurred mainly in TEs (Fig. 6c). Both DNA methylases and demethylases may regulate gene expression mainly by targeting TEs in gene promoter regions. However, DNA demethylase genes were not upregulated in *B. cinerea*-inoculated mulberry plants (Fig. S3). The role of DNA demethylase genes in mulberry disease resistance remains to be studied further. Instead, we detected the significant downregulation of multiple genes encoding DNA methyltransferases and other key components of the RdDM pathway (Fig. 2). The significant reduction in *MnMET1* transcript levels in *B. cinerea*-inoculated samples compared with mock samples suggested that MET1 is responsible for the significant reduction in mCG. In addition, passive DNA demethylation may occur when the maintenance methyltransferase is inactive during the cell cycle after DNA replication, resulting in the newly synthesized strand remaining in an unmethylated state. It is also possible that DNA demethylation activity is relatively increased owing to the decrease in DNA methyltransferase activity. This relative change may also be responsible for the declines in mCG and mCHG. In the RdDM pathway, 24-nt siRNAs direct DRM1 and DRM2 to methylate the CHH sequence context^10,43,44^. The significantly increased transcript levels of *MnRDR2* and *MnNRPD1* in *B. cinerea*-inoculated mulberry plants may explain the significant increase in mCHH. However, the abundance of 24-nt siRNAs was significantly decreased in *B. cinerea*-inoculated samples. Thus, the increase in mCHH in mulberry does not appear to be established and maintained only by the RdDM pathway. The downregulation of *MnDCL3* may be a main reason for the decrease in 24-nt siRNA levels. The abundance of 24-nt siRNAs in mulberry was closely related to CHG and CHH hypermethylation but less closely related to CG hypermethylation (Fig. 3d). There was no correlation between 24-nt siRNAs and CG hypermethylation in the *B. cinerea*-inoculated samples. In mulberry, many 24-nt siRNAs are derived from TEs, especially from the ends of TEs (Fig. 4b). This may be because long terminal repeats and terminal inverted repeats at both ends of TEs are more likely to form double chains. Among all the TEs, *MITE* was the main source of 24-nt siRNAs, further demonstrating the importance of *MITE*s in regulating gene expression through the RdDM pathway.

Promoter methylation is negatively correlated with gene expression levels^37,45–47^, but the correlation between gene body methylation and gene expression is less clear. Our results suggest that gene body methylation may be consistent with promoter region methylation, which is negatively correlated with gene expression (Fig. 6d). DNA hypomethylation of the promoters of nucleotide-binding leucine-rich repeat receptors enhances their expression levels, which subsequently improves resistance. For example, the promoter region of *Xa21G* was found to be hypomethylated in rice ‘Line-2’ and hypermethylated in wild-type rice. ‘Line-2’ showed high levels of gene expression and a resistant phenotype^48^. In *B. cinerea*-inoculated mulberry samples, two resistance-related genes, *Morus017734* and *Morus025913*, were hypomethylated in the gene body and promoter region, respectively, and their expression levels were significantly upregulated (Fig. 7). Treatment with 5-azacytidine significantly reduced the methylation levels of these two genes, which enhanced their expression and increased mulberry resistance to *B. cinerea*. In *Arabidopsis*, mutations in Argonaute 4 increase sensitivity to *Pseudomonas syringae*, whereas an *Arabidopsis* mutant deficient in maintaining mCG (*met1* mutant) showed enhanced resistance to *P. syringae*^5,49^. In mulberry, resistance to *B. cinerea* was increased by silencing *MnMET1* but not by silencing *MnAGO4* (Fig. 8). This occurred because MET1 maintains the CG methylation of resistance genes. In conclusion, our whole-genome DNA methylation analysis provided unprecedented insights into the changes in DNA methylation during the interaction between *B. cinerea* and mulberry. These changes affect a large number of protein-coding genes and their transcriptional regulation. Our results also showed that hypomethylation enhanced resistance to *B. cinerea* by inducing the expression of resistance genes. This is useful information for the methylation-targeted breeding of resistant varieties.

## Materials and methods

### Plant materials and the *B. cinerea* treatment

In total, 12 10-month-old mulberry (*M. notabilis* C.K. Schneid) seedlings were randomly divided into three replicates of two treatments as follows: inoculation with agar blocks without *B. cinerea* (mock-treated; mock) and inoculation with agar blocks containing *B. cinerea* (inoculated). The treated leaves on each plant were in the same position. Three days after the treatment, each whole-treated mulberry leaf was removed and sequenced. *B. cinerea* (MM1 isolate) was isolated from the mature mulberry fruit.

Five-month-old mulberry seedlings were treated with 20 mM of the DNA methylation inhibitor 5-azacytidine (Sigma, St. Louis, MO, USA) dissolved in ddH_2_0 with Triton X-100 (0.01%). The inhibitor was applied to the lower surfaces of leaves. ddH_2_0 with Triton X-100 (0.01%) was also applied to the lower surfaces of leaves in the control group. The treated leaves were wrapped with plastic wrap to prevent the solution from evaporating. This treatment was carried out on 10 June and again on 13 June, and the samples were inoculated with *B. cinerea* on 15 June.

### Genome-wide bisulfite sequencing and data analysis

Genomic DNA was extracted from leaves using the DNeasy plant Maxi Kit (Qiagen, Hilden, Germany). The samples were sequenced at the Beijing Genomics Institute (Shenzhen, China). The construction and sequencing of the bisulfite-seq library were performed as described previously^31,50^. The DNA was fragmented by sonication using a Bioruptor (Diagenode, Seraing, Belgium) into 250-bp fragments, on average; then, dA was added to the 3′ end and ligated with methylated adaptors. The EZ DNA Methylation-Gold kit (Zymo, Orange, CA, USA) was used to convert the ligated DNA. The fragments were sequenced using the Illumina HiSeq4000 platform (Illumina, San Diego, CA, USA). The data were mapped to the mulberry genome using BSMAP^51^. The parameters were set to -d ref.fa -u -v 9 -z 33 -p 8 -n 0 -w 20 -s 16 -f 10 -L 100.

The methylation level was determined by dividing the number of reads covering each mC by the total number of reads (≥ 4 reads effectively) covering that cytosine, which was also equal to the mC/C ratio at each reference cytosine^52,53^. The different methylated regions from different groups were calculated using metilene (v0.2-7)^54^. The parameters were set to -M 200 -m 5 -d 0.1 -t 1 -f 1 -X 2 -Y 2 -v 0.7. The significant regions were selected if they met the following criteria: mean methylation difference ≥ 0.1 and *p*-value ≤ 0.05. Gene element annotations of the methylated regions or DMRs from the different samples were performed. The GO (http://www.geneontology.org/) analyses and KEGG (https://www.kegg.jp/) enrichments of the DMR-related genes were also determined using phyper (https://en.wikipedia.org/wiki/Hypergeometric_distribution) to calculate p-values and determine the false discovery rate as assessed using the Bonferroni method. The details of the DMRs are listed in Table S2.

### RNA sequencing and data analyses

Ethanol precipitation and the CTAB-pBIOZOL reagent (Bioer, Hangzhou, China) were used to purify total RNA from plant tissues in accordance with the manufacturer’s instructions. The mRNA was purified using magnetic beads with attached oligo (dT)s. First-strand cDNA was generated by random hexamer-primed reverse transcription followed by second-strand cDNA synthesis. For RNA-seq, libraries were constructed and sequenced at the Beijing Genomics Institute using the BGIseq500 platform. The clean reads were mapped to the reference genome using HISAT2 (v2.0.4), and then the expression levels of genes were calculated using RSEM (v1.2.12). A differential expression analysis was performed using DESeq2 (v1.4.5) with a *Q*-value ≤ 0.05.

### Small-RNA sequencing and data analysis

TRIzol reagent (Ambion, Foster City, CA, USA) was used to extract total RNA from leaves. Small RNAs (~18–30 nt) were then isolated on denaturing polyacrylamide gels. The libraries were constructed and sequenced at the Beijing Genomics Institute using the BGIseq500 platform. Reads with 18 bp > lengths > 30 bp were discarded. The clean small RNA sequences were mapped to the genome using the anchor alignment-based small RNA annotation (AASRA) program. The mapped reads were normalized to the total clean reads for further analyses.

### Identification and phylogenetic analysis of DNA methyltransferases, DNA demethylases, and genes in the RdDM pathway of mulberry

BLASTn and BLASTp were performed with *Arabidopsis* and strawberry orthologs as queries against the mulberry genome database (https://morus.swu.edu.cn/morusdb/). The strawberry genome (https://phytozome.jgi.doe.gov/pz/portal.html#!info?alias=Org_Fvesca) and the *Arabidopsis* genome (https://www.arabidopsis.org) were used. The full-length cDNA sequences were aligned using ClustalW. The neighbor joining phylogenetic tree was constructed using MEGA6^55^.

### qRT-PCR analysis

qRT-PCR was performed using an Applied Biosystems StepOnePlus™ real-time PCR machine with the method described previously^20^. *MnActin* was used as the internal control for mulberry. Details about the qRT-PCR primers are listed in Table S3.

### McrBC-PCR analysis

McrBC-PCR detected the DNA methylation level within a target range. McrBC digestion was performed with 100-ng genomic DNA using the McrBC kit (NEB, Beijing, China) in accordance with the manufacturer’s instructions. The negative control was the digestion system without GTP. The primers were designed using Primer5 and are listed in Table S4.

### VIGS and transient overexpression

To investigate gene functions, VIGS based on TbCSV was used^56^. Fragments of *MnMET1* (428 bp), *MnAGO4* (419), and *MnPDS* (188 bp) were PCR-amplified from mulberry cDNA and cloned independently into the 2mDNA1 vector to generate the plasmids 2mDNA1-*MnMET1*, 2mDNA1-*MnAGO4*, and 2mDNA1-*MnPDS*, respectively. Mulberry mosaic dwarf-associated virus (MMDaV) was the auxiliary plasmid^57^. MMDaV, 2mDNA1, 2mDNA1-*MnMET1*, 2mDNA1-*MnAGO4*, and 2mDNA1-*MnPDS* were introduced independently into *Agrobacterium tumefaciens* strain GV3101. MMDaV and 2mDNA1 or their derivatives were mixed at 1:1 (v/v) ratios and infiltrated into the cotyledons of 1-week-old mulberry seedlings using a 1-cm^3^ syringe without a needle. The final optical density at a wavelength of 600 nm of each mixed *Agrobacterium* culture was 0.5. After 24 h in the dark, the mulberry seedlings were transferred to a greenhouse. At 14 days post agroinfiltration, the second true leaf was analyzed by qRT-PCR, and the first true leaf was inoculated with *B. cinerea*.

The full-length *MnMET1* coding sequence was cloned into the pLGNL vector between the *Kpn*I and *EcoR*I sites. Then, pLGNL and pLGNL-*MnMET1* were introduced independently into *A. tumefaciens* strain GV3101, which was then infiltrated into the leaves of 1-month-old mulberry seedlings. The primers used are listed in Table S5.

### Statistical analyses

All the graphs represent the results of multiple independent experiments (*n* ≥ 3), and the values are the means with standard deviations (SDs). All the data were analyzed using Excel 2013 (Microsoft, Seattle, WA, USA). A statistical significance analysis was performed using the two-tailed unpaired Student’s *t*-test, and a *P*-value < 0.05 was considered to indicate a statistically significant difference.

## Supplementary information

SUPPORTING INFORMATION

Table S1

Table S2

Table S3

Table S4

Table S5

## References

[1] Dean R (2012). The Top 10 fungal pathogens in molecular plant pathology. Mol. Plant Pathol..

[2] Legard DE, Xiao CL, Mertely JC, Chandler CK (2000). Effects of plant spacing and cultivar on incidence of Botrytis fruit rot in annual strawberry. Plant Dis..

[3] Angelini RMD (2014). Occurrence of fungicide resistance in populations of Botryotinia fuckeliana (Botrytis cinerea) on table grape and strawberry in southern Italy. Pest Manag. Sci..

[4] Lamichhane JR, Dachbrodt-Saaydeh S, Kudsk P, Messean A (2016). Toward a reduced reliance on conventional pesticides in European agriculture. Plant Dis..

[5] Dowen RH (2012). Widespread dynamic DNA methylation in response to biotic stress. Proc. Natl Acad. Sci. USA.

[6] Hewezi T, Howe P, Maier TR, Baum TJ (2008). Arabidopsis small RNAs and their targets during cyst nematode parasitism. Mol. Plant Microbe.

[7] Boyko A (2007). Transgenerational changes in the genome stability and methylation in pathogen-infected plants (virus-induced plant genome instability). Nucleic Acids Res..

[8] Yang SM, Tang F, Caixeta ET, Zhu HY (2013). Epigenetic regulation of a powdery mildew resistance gene in medicago truncatula. Mol. Plant.

[9] Zhu JK (2009). Active DNA demethylation mediated by DNA glycosylases. Annu. Rev. Genet..

[10] Law JA, Jacobsen SE (2010). Establishing, maintaining and modifying DNA methylation patterns in plants and animals. Nat. Rev. Genet..

[11] Zemach A (2013). The Arabidopsis nucleosome remodeler DDM1 allows DNA methyltransferases to access H1-containing heterochromatin. Cell.

[12] Matzke MA, Kanno T, Matzke AJ (2015). RNA-Directed DNA methylation: the evolution of a complex epigenetic pathway in flowering plants. Annu. Rev. Plant Biol..

[13] Matzke MA, Mosher RA (2014). RNA-directed DNA methylation: an epigenetic pathway of increasing complexity. Nat. Rev. Genet..

[14] Liu R, Lang ZB (2020). The mechanism and function of active DNA demethylation in plants. J. Integr. Plant Biol..

[15] Zhang H (2016). Transposon-derived small RNA is responsible for modified function of WRKY45 locus. Nat. Plants.

[16] Yoder JA, Walsh CP, Bestor TH (1997). Cytosine methylation and the ecology of intragenomic parasites. Trends Genet..

[17] Deng Y (2017). Epigenetic regulation of antagonistic receptors confers rice blast resistance with yield balance. Science.

[18] Tirnaz S, Batley J (2019). DNA methylation: toward crop disease resistance improvement. Trends Plant Sci..

[19] Jiang Y, Nie WJ (2015). Chemical properties in fruits of mulberry species from the Xinjiang province of China. Food Chem..

[20] Xin YC, Meng S, Ma B, He WM, He NJ (2020). Mulberry genes MnANR and MnLAR confer transgenic plants with resistance to Botrytis cinerea. Plant Sci..

[21] He N (2013). Draft genome sequence of the mulberry tree Morus notabilis. Nat. Commun..

[22] Ma B, Li T, Xiang Z, He N (2015). MnTEdb, a collective resource for mulberry transposable elements. Database.

[23] Xin YC, Ma B, Xiang ZH, He NJ (2019). Amplification of miniature inverted-repeat transposable elements and the associated impact on gene regulation and alternative splicing in mulberry (Morus notabilis). Mobile DNA.

[24] Song QX (2013). Genome-wide analysis of DNA methylation in soybean. Mol. Plant.

[25] Zakrzewski F, Schmidt M, Van Lijsebettens M, Schmidt T (2017). DNA methylation of retrotransposons, DNA transposons and genes in sugar beet (Beta vulgaris L.). Plant J..

[26] Huang H (2019). Global increase in DNA methylation during orange fruit development and ripening. Proc. Natl Acad. Sci. USA.

[27] Cheng J (2018). Downregulation of RdDM during strawberry fruit ripening. Genome Biol..

[28] Cokus SJ (2008). Shotgun bisulphite sequencing of the Arabidopsis genome reveals DNA methylation patterning. Nature.

[29] Zhang J (2015). Autotetraploid rice methylome analysis reveals methylation variation of transposable elements and their effects on gene expression. Proc. Natl Acad. Sci. USA.

[30] Zhong S (2013). Single-base resolution methylomes of tomato fruit development reveal epigenome modifications associated with ripening. Nat. Biotechnol..

[31] Xu J (2018). Single-base methylome analysis reveals dynamic epigenomic differences associated with water deficit in apple. Plant Biotechnol. J..

[32] Liang D (2014). Single-base-resolution methylomes of Populus trichocarpa reveal the association between DNA methylation and drought stress. BMC Genet..

[33] Ausin I (2016). DNA methylome of the 20-gigabase Norway spruce genome. Proc. Natl Acad. Sci. USA.

[34] Niederhuth CE (2016). Widespread natural variation of DNA methylation within angiosperms. Genome Biol..

[35] Hsieh TF (2009). Genome-wide demethylation of Arabidopsis endosperm. Science.

[36] Zemach A (2010). Local DNA hypomethylation activates genes in rice endosperm. Proc. Natl Acad. Sci. USA.

[37] Singer T, Yordan C, Martienssen RA (2001). Robertson’s mutator transposons in A. thaliana are regulated by the chromatin-remodeling gene decrease in DNA methylation (DDM1). Genes Dev..

[38] Biemont C, Vieira C (2006). Genetics: junk DNA as an evolutionary force. Nature.

[39] Wang C (2017). A transposon-directed epigenetic change in ZmCCT underlies quantitative resistance to Gibberella stalk rot in maize. New Phytol..

[40] Schumann U (2019). DEMETER plays a role in DNA demethylation and disease response in somatic tissues of Arabidopsis. Epigenetics.

[41] Schumann U, Lee J, Kazan K, Ayliffe M, Wang MB (2017). DNA-demethylase regulated genes show methylation-independent spatiotemporal expression patterns. Front. Plant Sci..

[42] Le TN (2014). DNA demethylases target promoter transposable elements to positively regulate stress responsive genes in Arabidopsis. Genome Biol..

[43] Cao X, Jacobsen SE (2002). Role of the arabidopsis DRM methyltransferases in de novo DNA methylation and gene silencing. Curr. Biol..

[44] Mosher RA, Melnyk CW (2010). siRNAs and DNA methylation: seedy epigenetics. Trends Plant Sci..

[45] Wang H (2015). CG gene body DNA methylation changes and evolution of duplicated genes in cassava. Proc. Natl Acad. Sci. USA.

[46] Zhang X (2006). Genome-wide high-resolution mapping and functional analysis of DNA methylation in arabidopsis. Cell.

[47] Jones PA (2012). Functions of DNA methylation: islands, start sites, gene bodies and beyond. Nat. Rev. Genet..

[48] Akimoto K (2007). Epigenetic inheritance in rice plants. Ann. Bot..

[49] Agorio A, Vera P (2007). ARGONAUTE4 is required for resistance to Pseudomonas syringae in Arabidopsis. Plant Cell.

[50] Lu X (2017). Single-base resolution methylomes of upland cotton (Gossypium hirsutum L.) reveal epigenome modifications in response to drought stress. BMC Genomics.

[51] Xi Y, Li W (2009). BSMAP: whole genome bisulfite sequence MAPping program. BMC Bioinform..

[52] Lister R (2009). Human DNA methylomes at base resolution show widespread epigenomic differences. Nature.

[53] Xiang H (2010). Single base-resolution methylome of the silkworm reveals a sparse epigenomic map. Nat. Biotechnol..

[54] Juhling F (2016). metilene: fast and sensitive calling of differentially methylated regions from bisulfite sequencing data. Genome Res..

[55] Tamura K, Stecher G, Peterson D, Filipski A, Kumar S (2013). MEGA6: molecular evolutionary genetics analysis version 6.0. Mol. Biol. Evol..

[56] Huang C, Xie Y, Zhou X (2009). Efficient virus-induced gene silencing in plants using a modified geminivirus DNA1 component. Plant Biotechnol. J..

[57] Ma Y (2015). Identification and molecular characterization of a novel monopartite geminivirus associated with mulberry mosaic dwarf disease. J. Gen. Virol..

