# Supplementary material for: Dynamic changes in transposable element and gene methylation in mulberry (Morus notabilis) in response to Botrytis cinerea
Source: Hortic Res. 2021 Jul 1;8:154. doi: 10.1038/s41438-021-00588-x (PMC8245511; doi:10.1038/s41438-021-00588-x)
Supplement: Supplementary file 1 — SUPPORTING INFORMATION [file 41438_2021_588_MOESM1_ESM.docx]

**
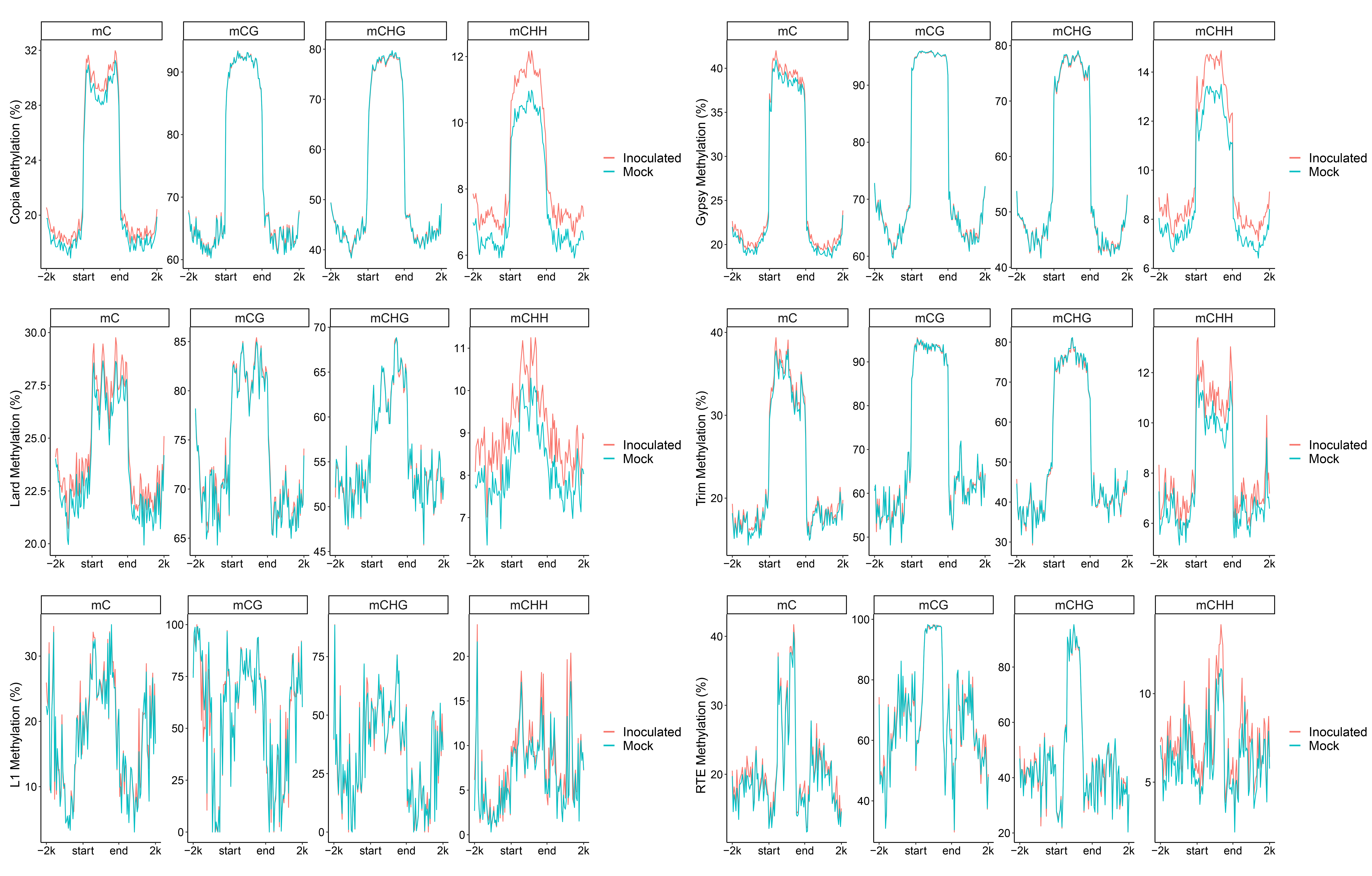
**

**Fig S1.** DNA methylation levels of mC, mCG, mCHG, and mCHH surrounding upstream regions, gene bodies, and downstream regions of retrotransposons in mock-treated (Mock) and *B. cinerea-*inoculated (Inoculated) mulberry leaves.


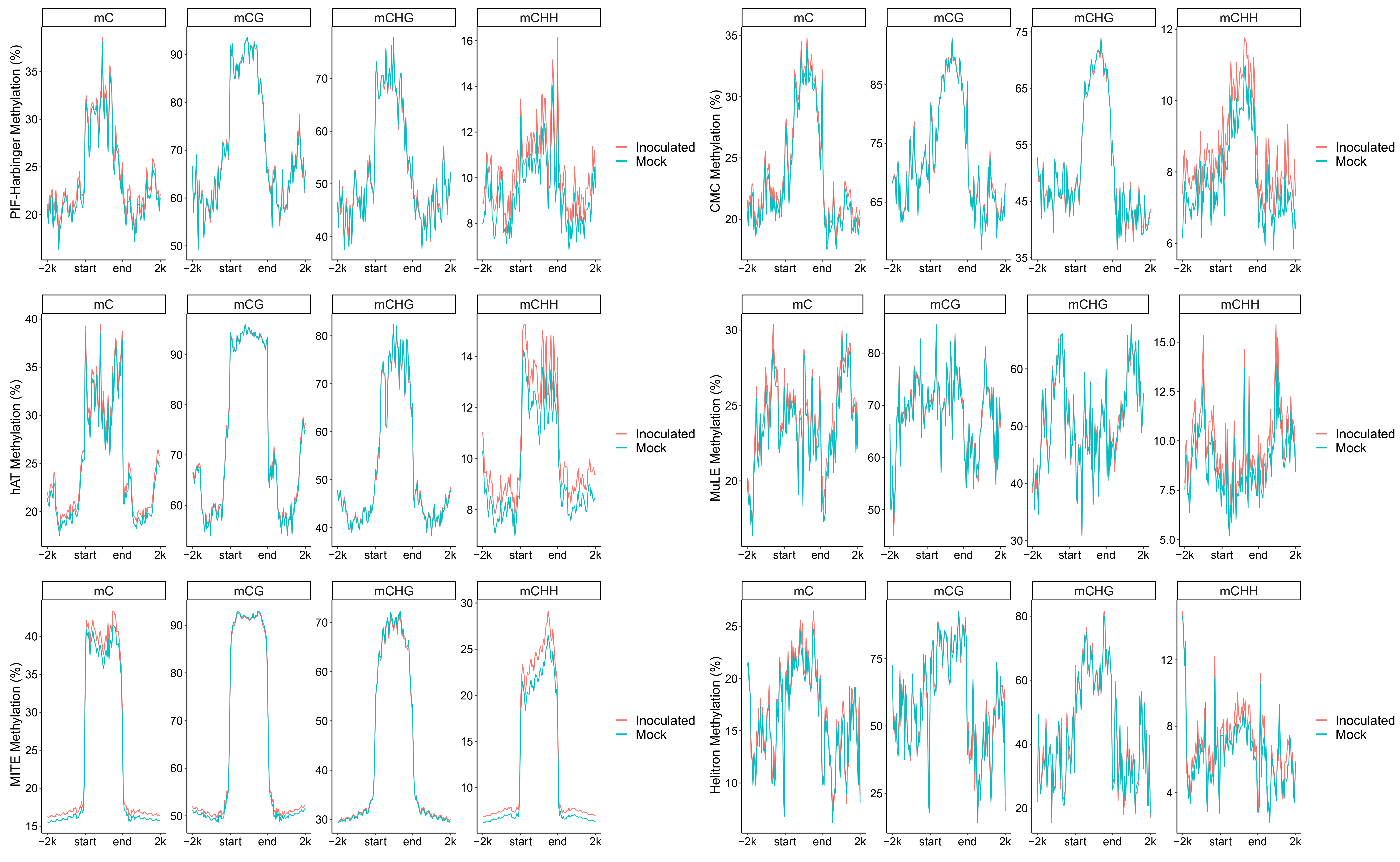


**Fig S2.** DNA methylation levels of mC, mCG, mCHG, and mCHH surrounding upstream regions, gene bodies, and downstream regions of DNA transposons in mock- treated (Mock) and *B. cinerea-*inoculated (Inoculated) mulberry leaves.


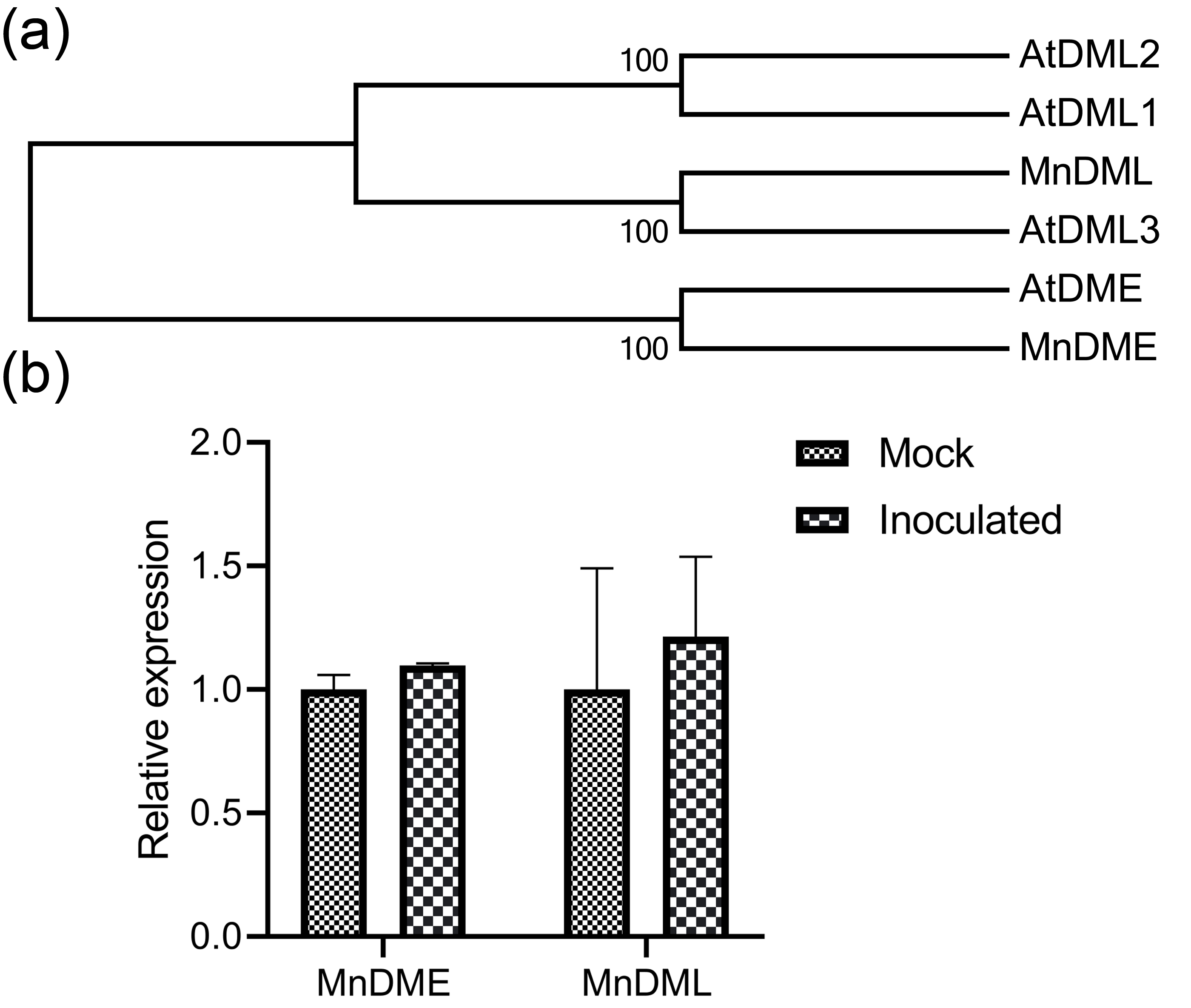


**Fig S3. Expression levels of genes involved in DNA demethylation.** **a** Phylogenetic analysis of genes involved in the DNA demethylation pathways of mulberry and *Arabidopsis*. The accession numbers of the CDSs are as follows: AtDML1 (AT2G36490), AtDML2 (AT3G10010), AtDML3 (AT4G34060), AtDME (AT5G04560), MnDME (KE346069.1), and MnDML (KE343785.1); **b** qPCR analyses of DNA demethylation genes. All the expression levels were normalized to the actin gene of mulberry. Error bars indicate SDs, n = 3 (**P*-value < 0.05, two-tailed *t*-test).


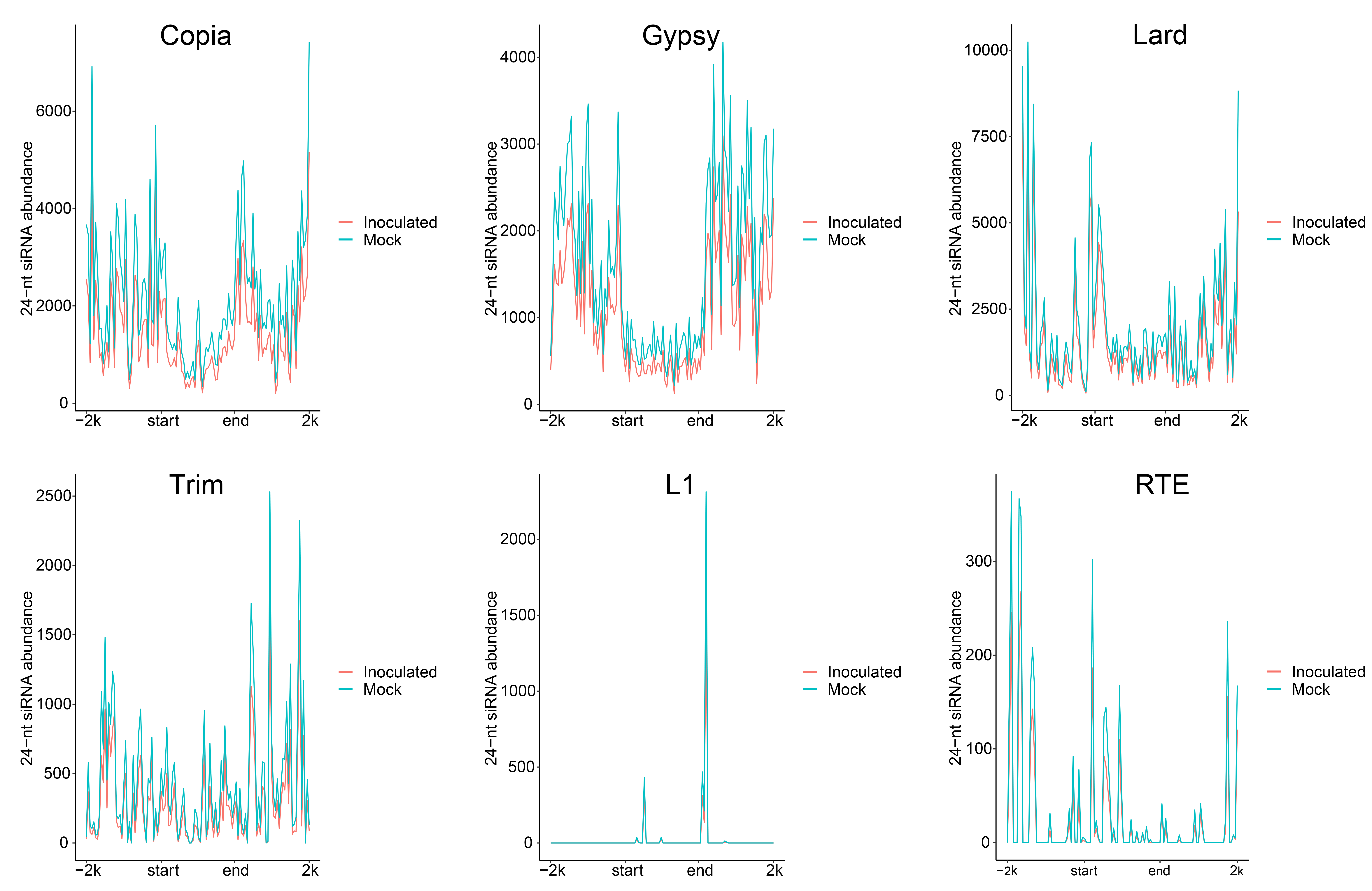


**Fig S4.** Distribution of 24-nt siRNAs surrounding upstream regions, gene bodies, and downstream regions of retrotransposons in mock-treated (Mock) and *B. cinerea-*inoculated (Inoculated) mulberry leaves.


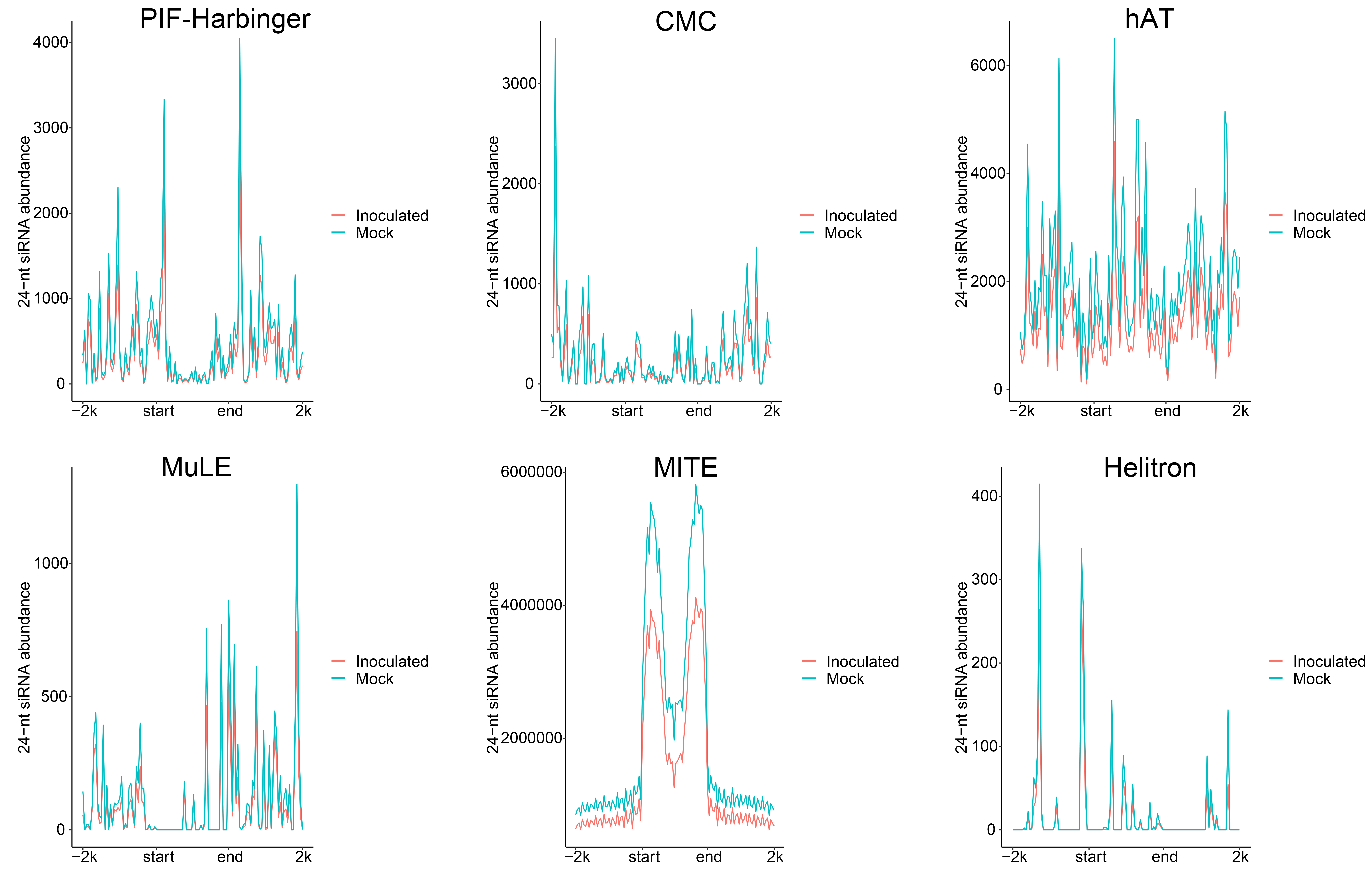


**Fig S5.** Distributions of 24-nt siRNAs surrounding upstream regions, gene bodies, and downstream regions of DNA transposons in mock-treated (Mock) and *B. cinerea-*inoculated (Inoculated) mulberry leaves.


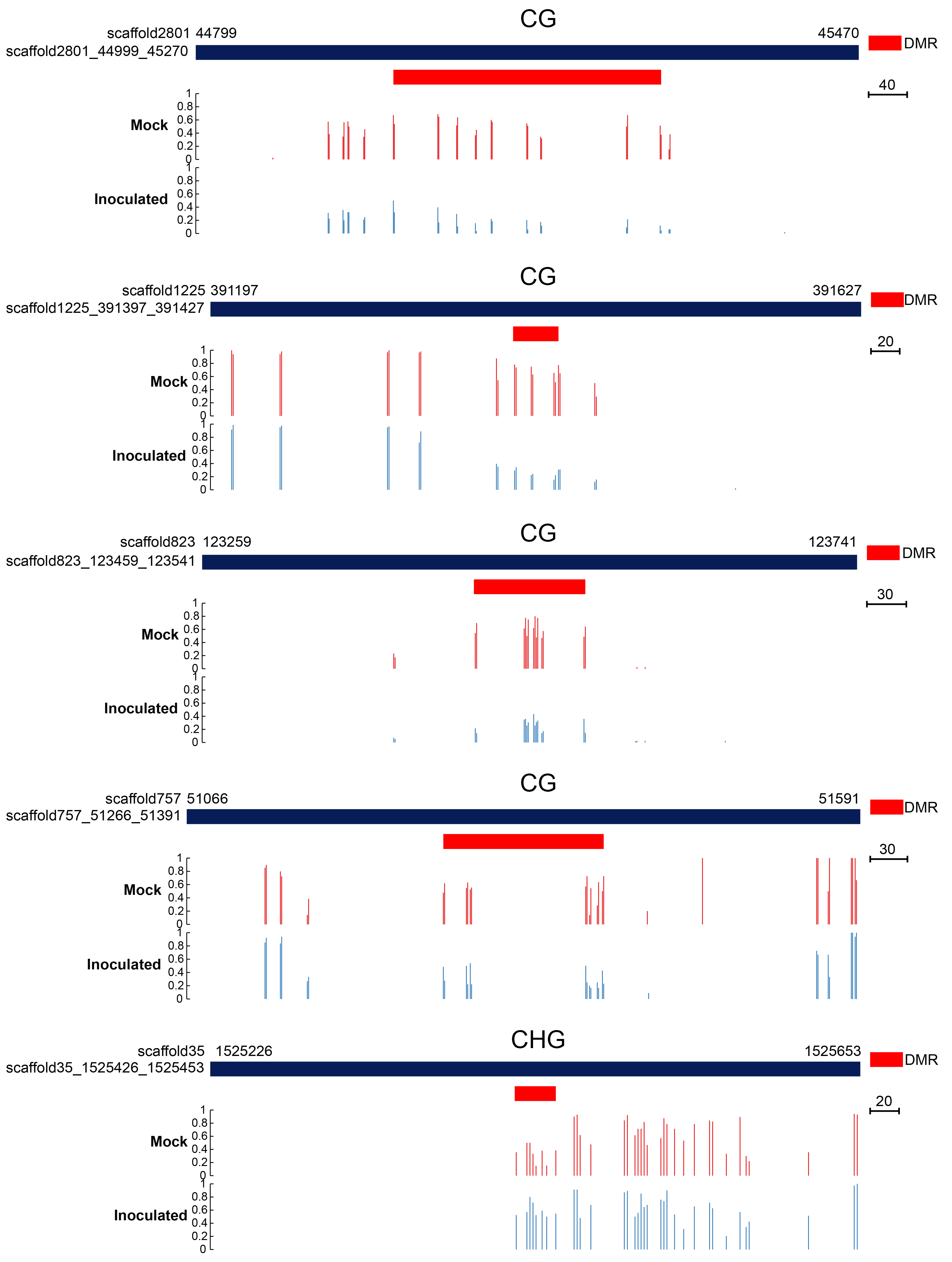


**Fig S6.** DNA methylation in mock-treated (Mock) and *B. cinerea-*inoculated (Inoculated) mulberry leaves. The red rectangles represent DMRs.


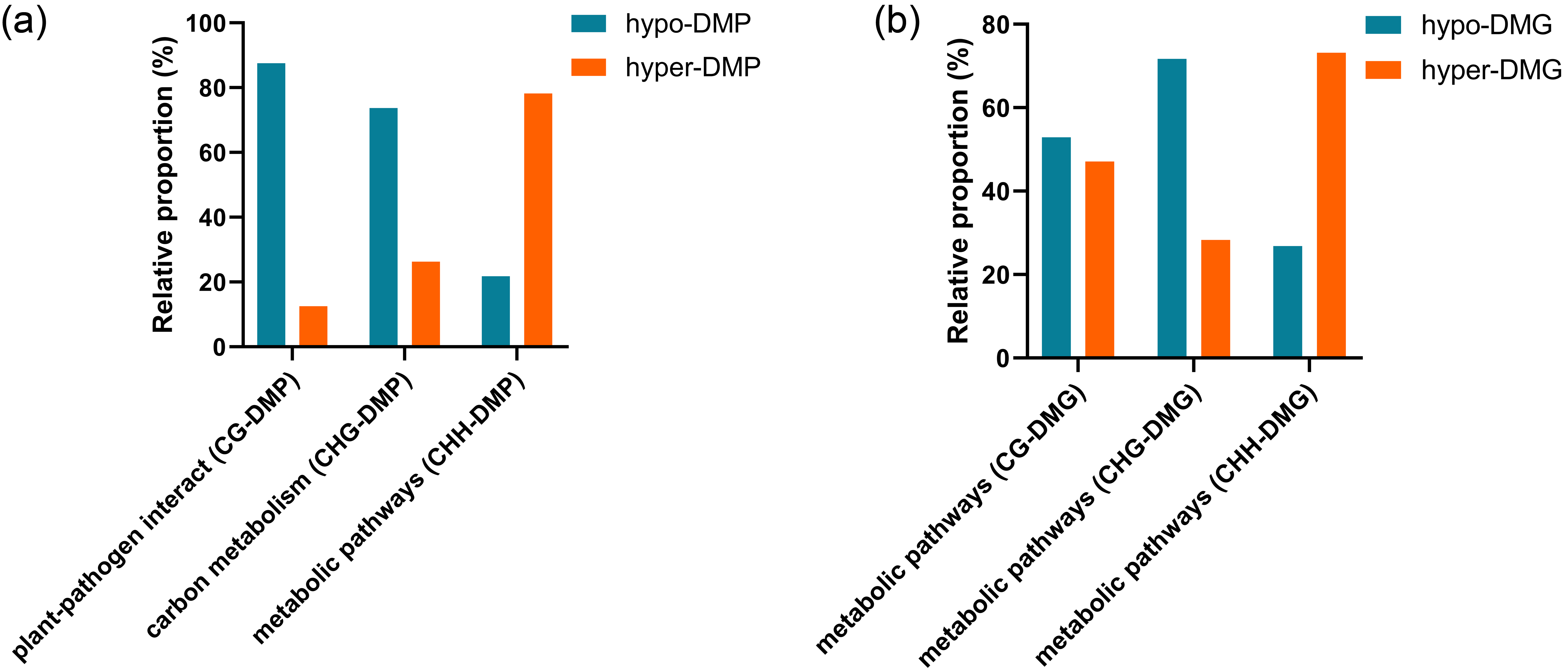


**Fig S7.** The relative proportions of hypomethylation and hypermethylation in three classes of genes enriched in KEGG pathways. **a** differentially methylated promoters; and **b** differentially methylated genes.


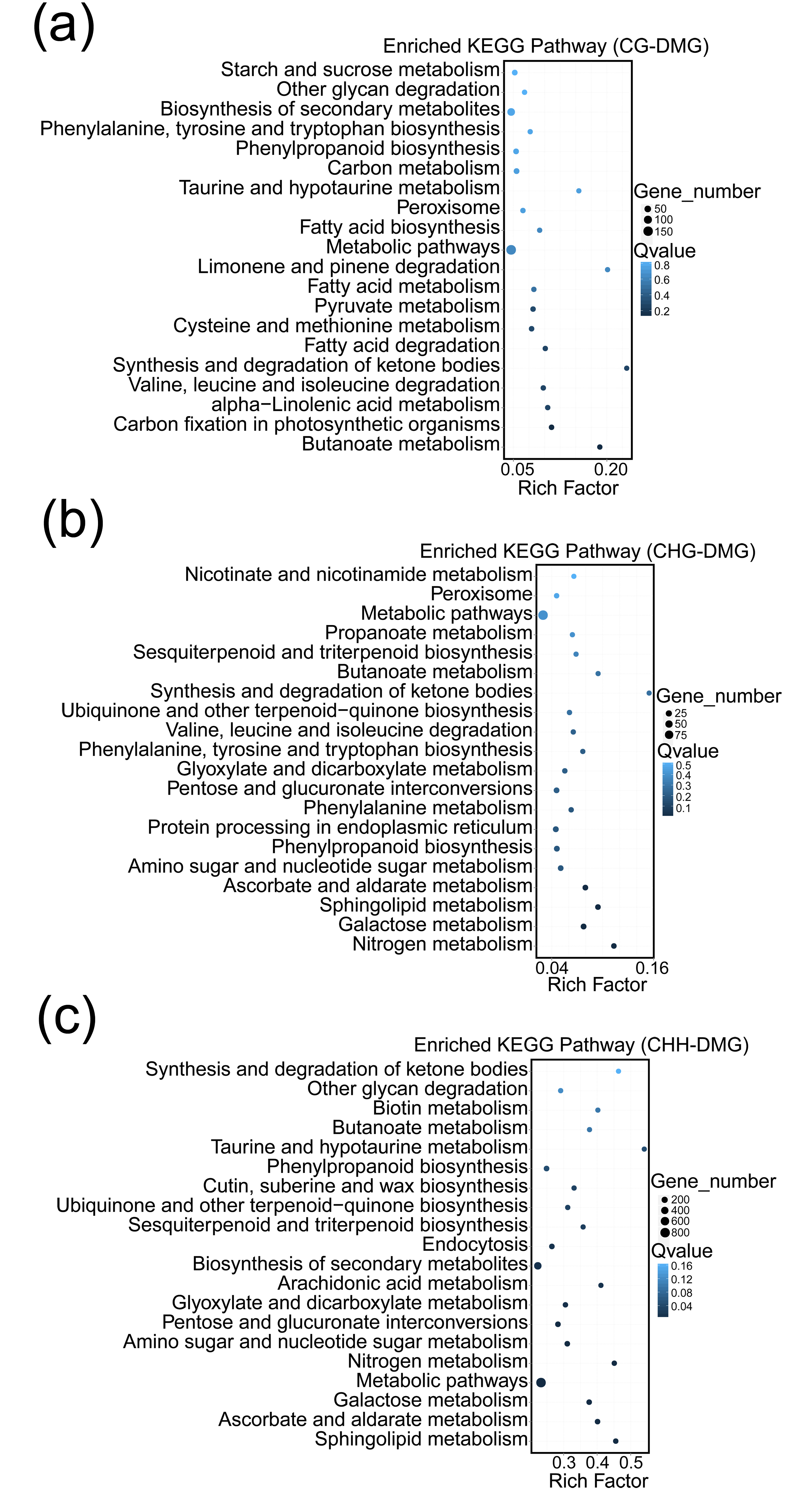


**Fig S8.** KEGG pathway enrichment analyses of differentially methylated genes. **a** CG; **b** CHG; and **c** CHH methylation.


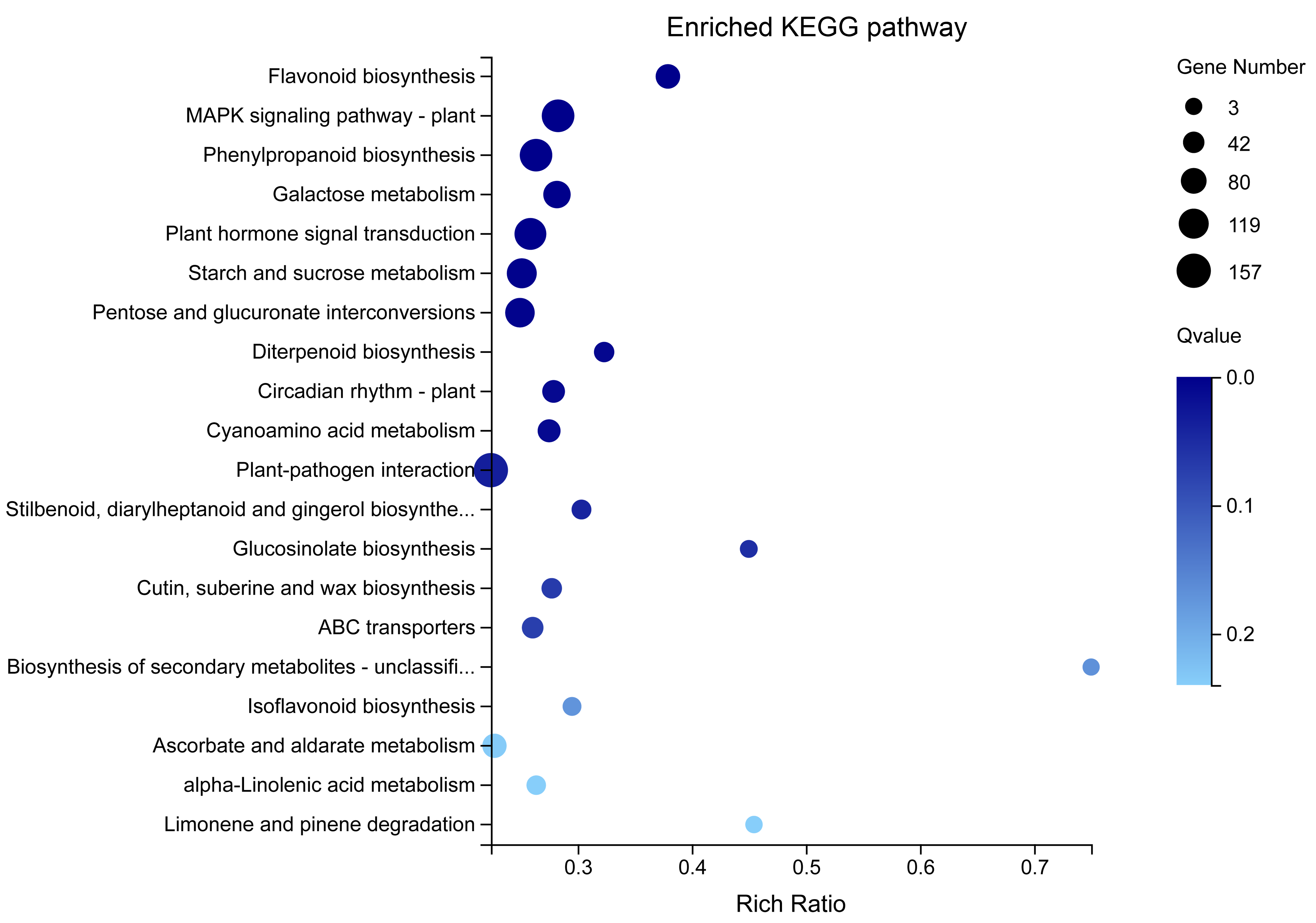


**Fig S9.** KEGG pathway enrichment analyses of differentially expressed genes in the transcriptome data.


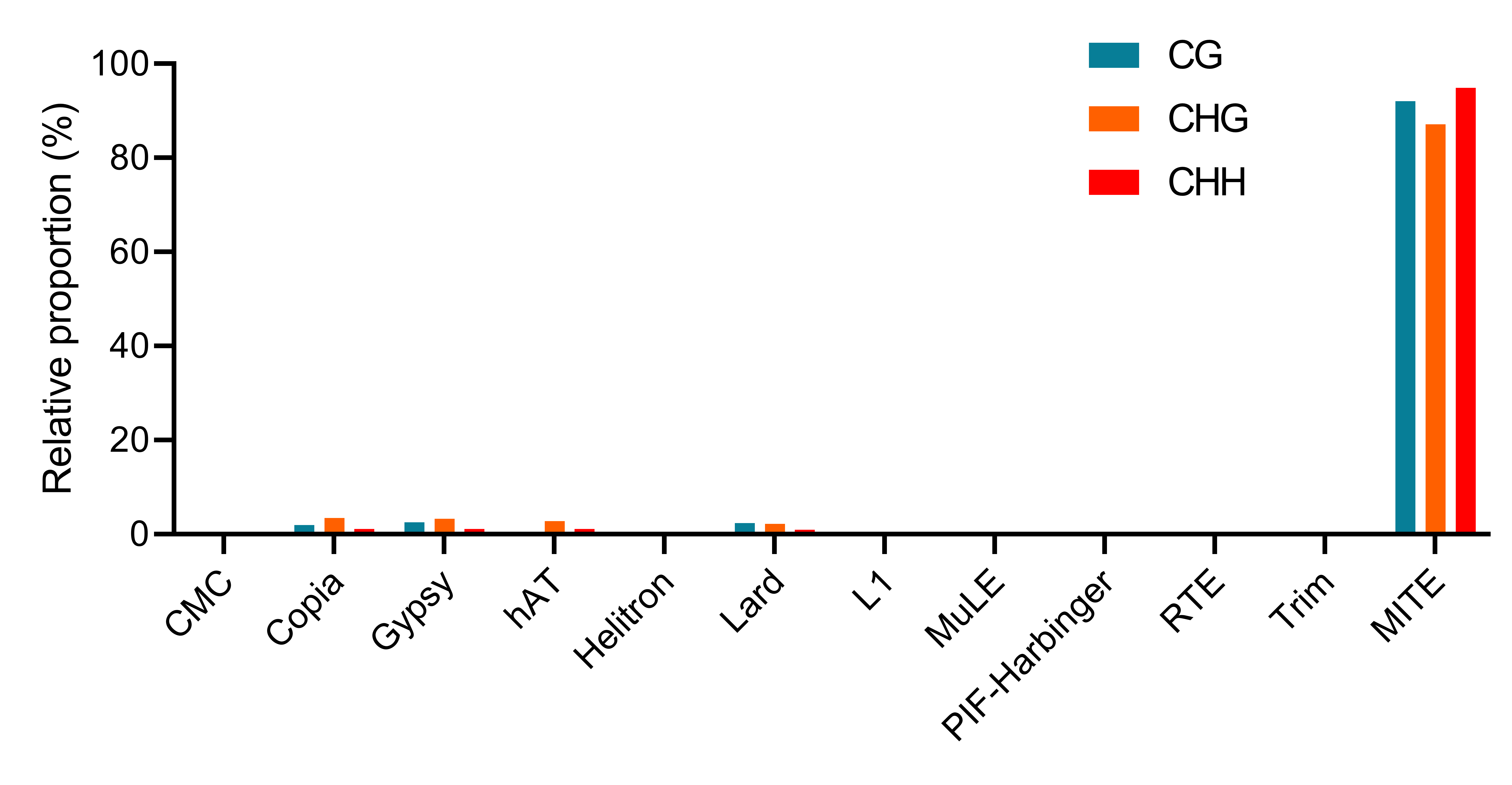


**Fig S10.** The proportions of differentially methylated promoters overlapping TEs in each superfamily.


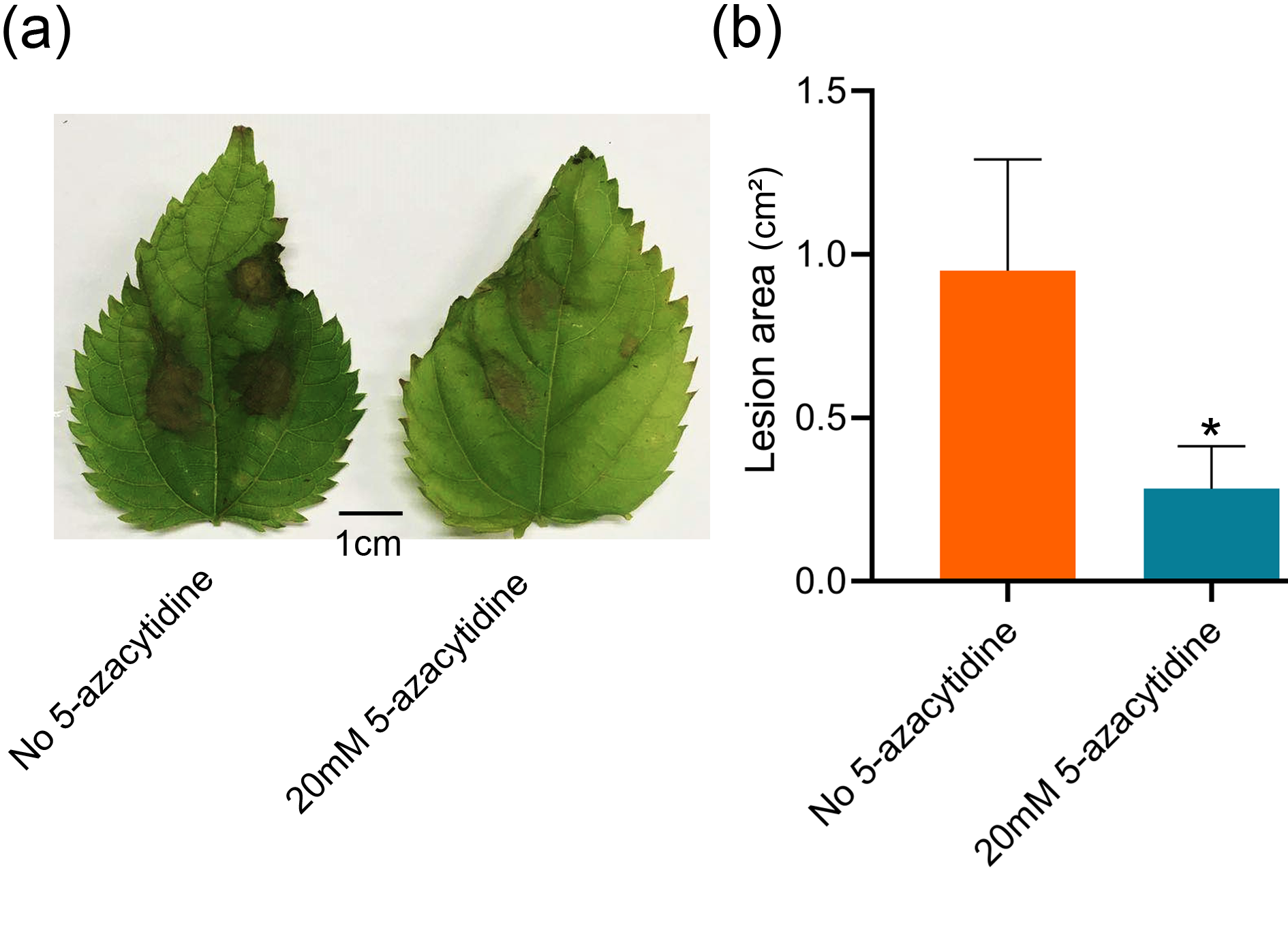


**Fig S11.** **Resistance of 5-azacytidine-treated mulberry leaves inoculated with *Botrytis cinerea*.** **a** Mulberry leaves inoculated with *B. cinerea* were photographed at 3 days after the inoculation; **b** Quantitative resistance analyses of mulberry leaves treated with and without 5-azacytidine after infection.


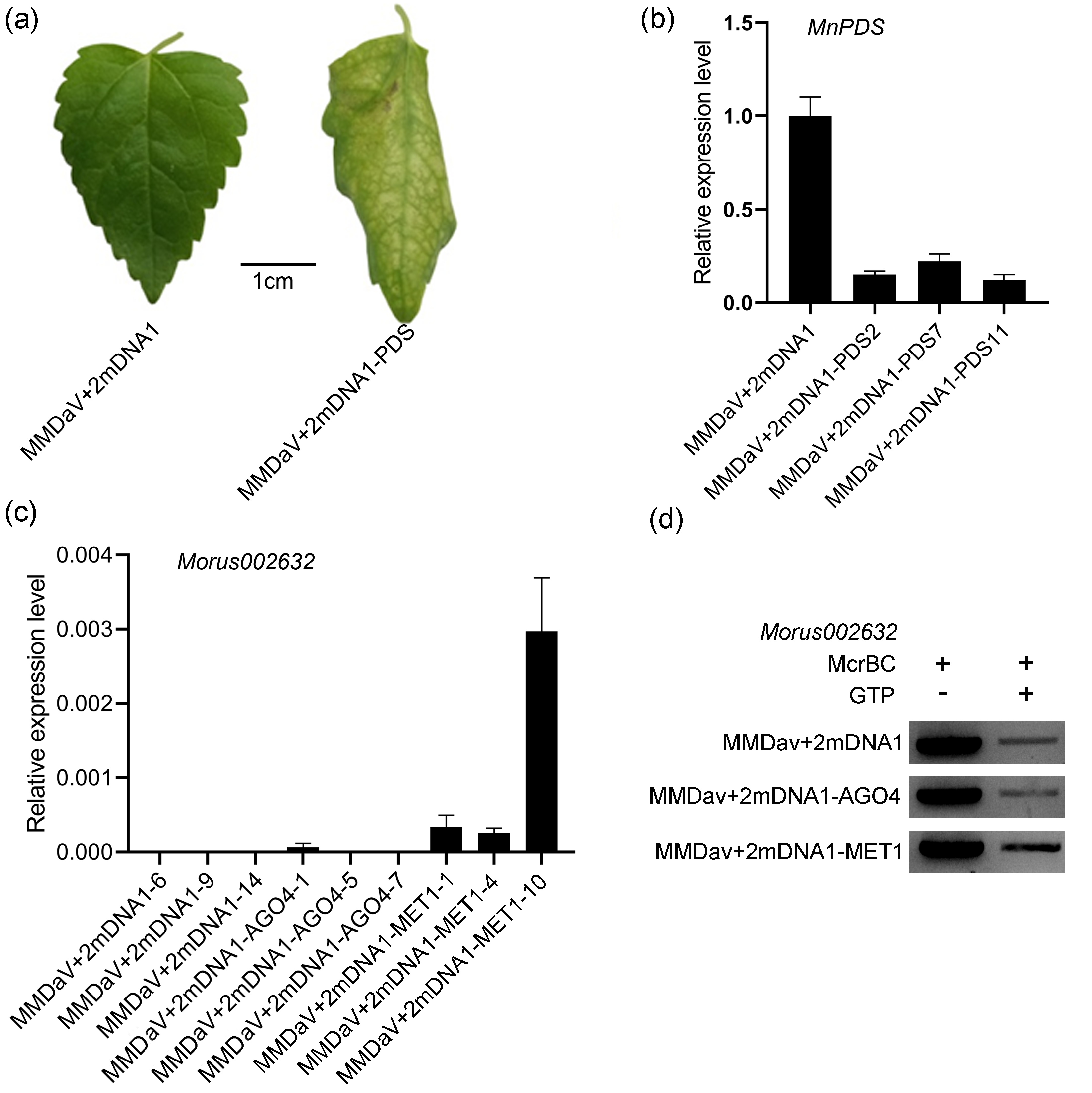


**Fig S12. Silencing of *MnPDS* in mulberry leaves resulted in color reduction.** **a** Mulberry leaves injected with MMDaV+2mDNA1 and MMDaV+2mDNA1-PDS; **b** Relative expression of *MnPDS* in leaves injected with MMDaV+2mDNA1 and MMDaV+2mDNA1-PDS; **c** *Morus002632* expression in leaves injected with MMDaV+2mDNA1, MMDaV+2mDNA1-AGO4, and MMDaV+2MDNA1-MET1; **d** McrBC-PCR analysis of *Morus002632*. + and − indicate the presence and absence of GTP, respectively. All the expression data were normalized against the expression level of the mulberry actin gene. Error bars indicate SDs, n = 3.


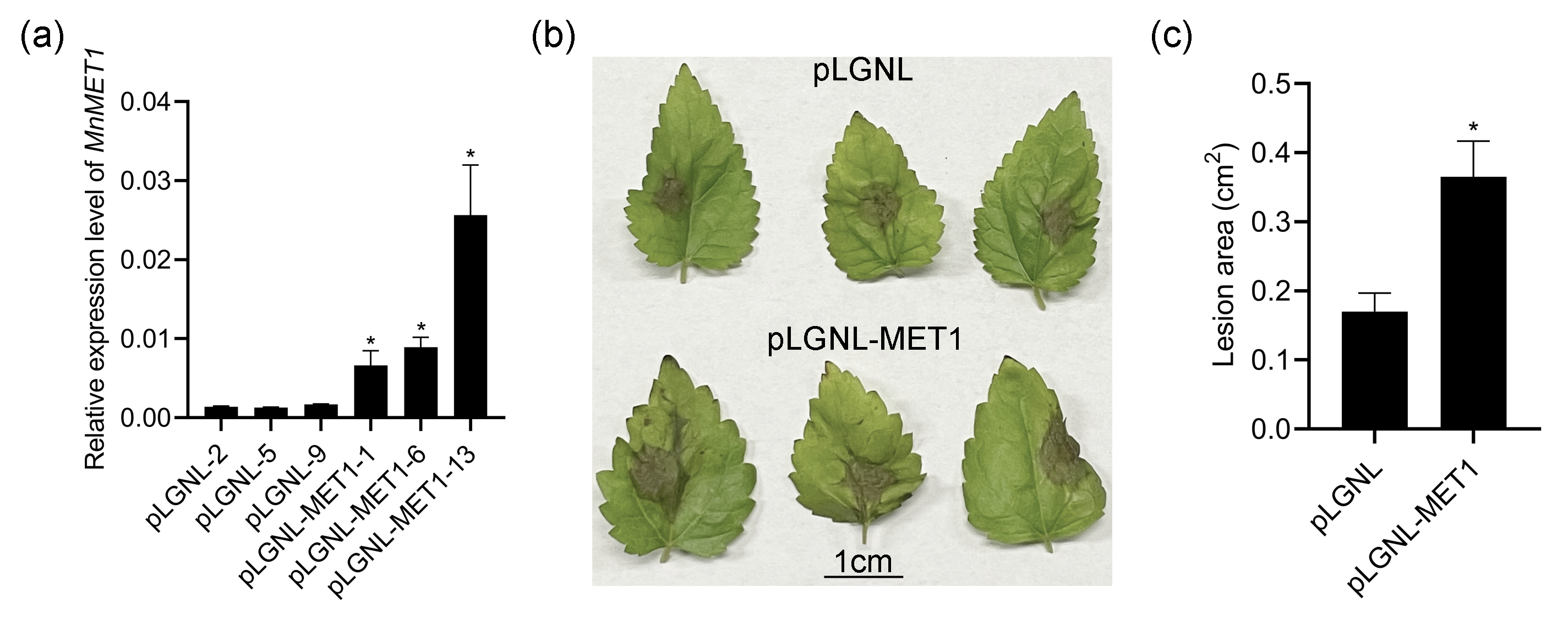


**Fig S13. Transient overexpression of *MnMET1* in mulberry leaves resulted in reduced resistance. a** Relative expression levels of *MnMET1* in mulberry leaves collected from three replicates injected with pLGNL and pLGNL-MET1. All the expression levels were normalized to that of the mulberry actin gene. Error bars indicate SDs, n = 3 (**P*-value < 0.05, two-tailed *t*-test). **b** Mulberry leaves inoculated with *B. cinerea* were photographed at 2 days after inoculation. **c** Quantitative analyses of the resistance levels of mulberry leaves injected with pLGNL and pLGNL-MET1.
