# Supplementary material for: Dynamic changes in transposable element and gene methylation in mulberry (Morus notabilis) in response to Botrytis cinerea
Source: Hortic Res. 2021 Jul 1;8:154. doi: 10.1038/s41438-021-00588-x (PMC8245511; doi:10.1038/s41438-021-00588-x)
Supplement: Supplementary file 4 — Table S3 [file 41438_2021_588_MOESM4_ESM.docx]

Table S2. Primers for real-time PCR.

| **Gene ID** | **Gene symbol** | **Forward primer** | **Reverse primer** |
| --- | --- | --- | --- |
| *Morus017734* |  | TTGGTGCGATCTGGAGCCT | CGACTTTAGCATCTGAAGC |
| *Morus025913* |  | TAACCTAACAGGCGAGAT | AAGGCTTCCAGACAAACT |
| *Morus002632* |  | TCAAGGCTGAATGCGAAAC | GATAATCCAATGCCGATGC |
| *Morus010063* | *MnPDS* | ATACTTGGTGGCCAGCCTTA | CCCTTCTATCGCACTTCCAT |
| *Morus003731* | *MnMET1* | CAAGCGACGACGACACGACT | TAGCAGCATTGCCGAGAACC |
| *Morus022822* | *MnCMT2* | TTCTGGAAACTGCGACTC | TTCAAGGGCTTAGGTGCT |
| *Morus006523* | *MnCMT3* | GGCTTTAACCGTTACAGGA | GTAGGCACCAGCAGGCATC |
| *Morus020526* | *MnDRM1* | GCAGCCAGAGGGTTAGAC | AGCATACTTTGCGTCCTG |
| *Morus026137* | *MnDRM3* | TCATTGTTGCTGCCCAGATAG | AACAGGAGTCCGACCCGTAAA |
| *Morus022517* | *MnAGO4* | ATGCTGAGCCAATGTTAC | GCCCACCTTTCTATCTTA |
| *Morus009825* | *MnAGO6* | TGAAATCCATTGCCCTTGC | CGCCTGCTGCCTCAGAATA |
| *Morus018624* | *MnDCL3* | CGATTTCTATTGCGTCTC | AAACTCAAGCCTCTGGTA |
| *Morus023971* | *MnNRPE1* | TCTAAAGGCGAGCCCGATAA | CAGGAGTGGCATCCCAAACA |
| *Morus002118* | *MnRDR2* | TGCTTGGACGAAACTGGG | GCCGCCTAAGGTGTCATT |
| *Morus023130* | *MnNRPD1* | CGGAGCAGGCATGTATGT | CAGATTTGGGAGCGTCAG |
| *Morus016801* | *MnDME* | GGGAATCCGAATGCTAATGC | GAACCTGAACTGAGACCACC |
| *Morus020735* | *MnDML* | GCTCTGCCTTTATGTCCCTT | TGTCCTCCTTAGTTCGTCCC |
|  | *MnActin* | GCATGAAGATCAAGGTGGTG | CATCTGCTGGAAGGTGCTAA |
