# Supplementary material for: Dynamic changes in transposable element and gene methylation in mulberry (Morus notabilis) in response to Botrytis cinerea
Source: Hortic Res. 2021 Jul 1;8:154. doi: 10.1038/s41438-021-00588-x (PMC8245511; doi:10.1038/s41438-021-00588-x)
Supplement: Supplementary file 5 — Table S4 [file 41438_2021_588_MOESM5_ESM.docx]

Table S3. Primers for McrBC-PCR.

| **Gene ID** | **Forward primer** | **Reverse primer** |
| --- | --- | --- |
| *Morus017734* | CGATATCTTTGCCCAAACTATCC | GACATATGAGTCGACTGCTCA |
| *Morus025913*  *Morus002632* | AAAAGACTACGTGATTGCG  ACAACCACCGTATTCCTCAC | AGGTTTAGTCCCGTTCATT  CCGTAGAGCAATAACGAAAG |
