# Supplementary material for: Dynamic changes in transposable element and gene methylation in mulberry (Morus notabilis) in response to Botrytis cinerea
Source: Hortic Res. 2021 Jul 1;8:154. doi: 10.1038/s41438-021-00588-x (PMC8245511; doi:10.1038/s41438-021-00588-x)
Supplement: Supplementary file 6 — Table S5 [file 41438_2021_588_MOESM6_ESM.docx]

Table S4. Primers for the construction of VIGS and overexpression vectors.

| **Gene ID** | **Gene symbol** | **Forward primer** | **Reverse primer** |
| --- | --- | --- | --- |
| *Morus003731* | *MnMET1* | GCTCTAGACATTGGCACGGGCAACACCA | CGGGATCCTGATGAATCCCGCCCCAACAG |
| *Morus022517* | *MnAGO4* | GCTCTAGATTCTGCCTAGATTTCTCCACCA | CGGGATCCCAACCTGGCCCTGTTGTGGA |
| *Morus010063* | *MnPDS* | GCTCTAGACTTGGTGTTTTTACAACATG | CGGGATCCGAATATTACAGTCCAAACCA |
|  | *MnMET1* | TTTCATTTGGAGAGGACAGGGTACCAT  GGGATCTGTAGAGCTTTTGGA | TCTCATTAAAGCAGGGAATTCCTAGCTC  GACCTCTTACTGTC |
